# Associations of adiposity and weight change with recurrence and survival in breast cancer patients: a systematic review and meta-analysis

**DOI:** 10.1007/s12282-022-01355-z

**Published:** 2022-05-17

**Authors:** Yuanjie Pang, Yuxia Wei, Christiana Kartsonaki

**Affiliations:** 1 Department of Epidemiology and Biostatistics, School of Public Health, Peking University, 38 Xueyuan Road, Beijing, 100191 China; 2 Institute of Environmental Medicine, Karolinska Institutet, C6 Institutet för miljömedicin, 17177 Stockholm, Sweden; 3 Clinical Trial Service Unit & Epidemiological Studies Unit (CTSU), Nuffield Department of Population Health, University of Oxford, Big Data Institute Building, Roosevelt Drive, Oxford, UK; 4 Medical Research Council Population Health Research Unit (MRC PHRU), Nuffield Department of Population Health, University of Oxford, Big Data Institute Building, Old Road Campus, Oxford, OX3 7LF UK

**Keywords:** Adiposity, Weight change, Breast cancer survival, Systematic review, Meta-analysis

## Abstract

**Background:**

Adiposity and weight change among patients with breast cancer are associated with mortality, but there is limited evidence on the associations with distant recurrence or other causes of death or on central adiposity. Moreover, the relationship with breast cancer subtypes and by menopause status is unclear.

**Methods:**

We conducted a systematic review and meta-analysis of prospective studies of breast cancer patients investigating the associations of general and central adiposity (body mass index [BMI] and waist circumference [WC], respectively), before and after diagnosis, and weight change, with all-cause mortality, breast cancer-specific mortality (BCSM), and recurrence.

**Results:**

173 studies (519,544 patients, 60,249 deaths overall, and 25,751 breast cancer deaths) were included. For BMI  < 1 year post diagnosis, compared with normal weight women, the summary relative risk (RR) for obese women was 1.21 (1.15–1.27) for all-cause mortality, 1.22 (1.13–1.32) for BCSM, 1.12 (1.06–1.18) for recurrence, and 1.19 (1.11–1.28) for distant recurrence. Obesity was associated with all-cause mortality and BCSM in patients with ER+ or HER2+ tumors, whereas no clear association was observed in patients with triple-negative tumors. Similar associations were observed by menopausal status. Stronger associations were observed in East Asians than Europeans. Central adiposity was associated with all-cause mortality, while large weight gain was associated with all-cause mortality, BCSM, and recurrence.

**Conclusion:**

Higher adiposity is associated with all-cause mortality, BCSM, recurrence, and distant recurrence in breast cancer patients, with similar associations by menopausal status and some evidence of heterogeneity by subtypes. Weight gain is also associated with recurrence and survival among breast cancer patients.

**Supplementary Information:**

The online version contains supplementary material available at 10.1007/s12282-022-01355-z.

## Introduction

Breast cancer has overtaken lung cancer as the most commonly diagnosed cancer worldwide [1]. There were 2.3 million cases of breast cancer and over 680,000 deaths among females worldwide in 2020 [2]. Despite advances in treatment in recent decades and earlier detection due to screening and improvements in prediction of breast cancer risk, a substantial proportion of breast cancer patients have recurrence of their disease at distant sites and subsequently die of their disease. The risks of distant metastasis and death after treatment of operable early or locally advanced breast cancer vary greatly by tumor subtype and patient characteristics [3].

Previous systematic reviews and meta-analyses have shown that overweight and obesity before or shortly after diagnosis [4, 5], as well as weight gain after diagnosis [5], are associated with higher risks of breast cancer-specific mortality (BCSM), all-cause mortality, and recurrence. A Mendelian randomization study showed that higher adiposity was associated with lower survival in estrogen receptor (ER)-positive but not in ER-negative breast cancer patients [6]. Randomized controlled trials of weight loss interventions have been conducted [7, 8], but no definitive evidence yet exists on whether these improve survival or reduce recurrence risk [9].

Several potential mechanisms for the associations between adiposity and breast cancer outcomes have been proposed. In post-menopausal women, the synthesis of estrogens from androgens in adipose tissue is a primary source of circulating estrogens, and overweight and obesity have been shown to be associated with higher estrogen levels [10, 11]. Higher levels of insulin and interactions between cytokines, hormones and markers of inflammation may contribute [12, 13]. Moreover, obesity may adversely impact outcomes due to chemotherapy dose capping for obese patients to limit toxicity, resulting in lower dose intensity. A recent meta-analysis showed that among breast cancer patients who received neoadjuvant chemotherapy, overweight and obese patients had a lower pathological complete response (pCR) rate compared to those with under- or normal weight [14]. Biological effects are plausible as an association of higher levels of adiposity with risk of developing breast cancer in the first place among post-menopausal women is well established [15].

There is uncertainty on the extent to which the associations of various adiposity features with outcomes vary by menopause status, tumor subtype or other patient or tumor characteristics. We conducted a systematic review and meta-analysis to assess the associations of adiposity or change in adiposity near the time of diagnosis with survival and risk of recurrence among patients with early or operable locally advanced breast cancer, overall, and by tumor and patient characteristics.

## Materials and methods

### Search strategy

A systematic literature search was conducted without language restrictions for articles on adiposity, weight change, and recurrence or survival in breast cancer patients in EMBASE and PubMed from inception to 1 October 2020. The search strategy is available in Supplementary Methods. In addition, we searched the reference lists of original articles, reviews, and meta-analyses. Searches were re-run on 1 April 2021 to include additional studies published prior to the final analysis. The protocol is registered at PROSPERO (CRD42020214730) and is available at https://www.crd.york.ac.uk/prospero/display_record.php?ID=CRD42020214730.

### Study selection and data extraction

Eligible studies were prospective cohort studies, randomized controlled trials (RCTs), other non-randomized trials of breast cancer patients, or case series with more than 50 patients, which reported estimates of the associations of adiposity assessed before and after breast cancer diagnosis or changes in adiposity with breast cancer-specific or all-cause mortality, recurrence, or metastasis, among adults with early or operable locally advanced breast cancer who receive treatment with curative intent. We excluded retrospective studies, reviews, conference abstracts which did not report sufficient data, and studies on patients with inoperable of metastatic breast cancer (studies in which only a small proportion of patients had metastatic breast cancer were initially included and a sensitivity analysis excluding them was done). When multiple publications on the same study population were found, results based on longer follow-up and more cases were selected for the meta-analysis.

The titles and abstracts identified by the search were screened by one author (YP) in collaboration with two librarians. Duplicates and articles which did not meet the inclusion criteria were removed. The full texts of all studies identified as being potentially eligible for inclusion were then obtained and assessed by two review authors (YP and YW), who independently screened and assessed their eligibility for inclusion. Disagreements were addressed by discussion between the two authors, with any remaining differences resolved by recourse to a third review author (CK). Study characteristics and results were extracted using a data extraction form by two review authors (YP and YW) (Supplementary Methods).

### Comparisons and outcomes

For adiposity, studies that investigated general adiposity (measured by BMI), central adiposity (waist circumference, waist-to-hip ratio [WHR]), or hip circumference, or weight change were included. According to BMI assessment period, studies were assigned to one of three groups: pre-diagnosis (including at the time of diagnosis), < 1 year after diagnosis, and 1 year or more after diagnosis.

In the majority of the included studies, the reference category was normal weight or underweight/normal weight according to the World Health Organization international classification with slightly different cut-off points used in some studies. The main analyses were conducted for underweight, overweight, obese, and morbidly obese compared with normal weight or underweight/normal weight and for obese compared with non-obese. The majority of studies separated overweight and obese, while some studies combined overweight and obese. Estimates from studies with underweight as the reference were converted such that they could be included in the meta-analysis. Studies which compared high vs low categories without specifying cut-off points were excluded.

For central adiposity, the main analyses were conducted comparing high vs low categories because studies used different cut-off points. In secondary analyses, centrally obese vs non-obese were compared using standard cut-off points (e.g., 80 cm for WC).

Weight change was calculated as the difference between post-diagnosis weight and weight prior to or at diagnosis. The reference category was weight maintenance (< ± 5% change). Three comparisons were conducted: 1) moderate weight gain (5–10%), 2) high weight gain (> 10%), and 3) any weight gain (> 5%). Some studies used slightly different cut-off points for moderate (4–6%) and high weight gain (8–12%).

Primary outcomes included all-cause mortality, BCSM, recurrence, and distant metastasis. Secondary outcomes included loco-regional recurrence, disease-free survival (DFS), and recurrence-free survival (RFS). RFS was combined with DFS because the majority of included studies did not specify how invasive contralateral breast cancer and secondary primary invasive cancer (non-breast) were handled according to STEEP definitions [16].

### Risk of bias

For randomized studies, we evaluated the risk of bias based on the Cochrane Collaboration ‘Risk of bias’ tool (‘high risk of bias’, ‘low risk of bias’, or ‘unclear’). For non-randomized studies, we used a modified Newcastle–Ottawa quality assessment scale, including eight items with nine scores. All included studies were accessed by two authors (YP and YW) independently. Publication bias was examined by Egger’s test and visual inspection of the funnel plots.

### Statistical analysis

We conducted meta-analyses using a random effects model. The maximally adjusted RR estimates were used for the meta-analysis except for estimates with additional adjustment for BMI or central adiposity.

We also conducted linear dose–response meta-analyses to examine the RR per 1 kg/m^2^ higher BMI in relation to overall mortality, using the method described by Greenland and Longnecker (which allows for non-independence of relative risk estimates within each study) [17]. As previous meta-analysis reported a J-shaped association between BMI and all-cause mortality among breast cancer patients, underweight was excluded from the dose–response meta-analysis. We then pooled the estimated linear trends using inverse-variance weighted fixed effects meta-analysis along with previous studies reporting RR per unit increase. Dose–response meta-analysis included studies reporting at least 3 BMI categories. When the extreme BMI categories were open-ended, the width of the adjacent close-ended category was used to estimate the midpoints.

To assess heterogeneity, we used the *I*^2^ statistic and the associated *χ*^2^ test for heterogeneity [18]. The cut points of 30% and 50% were used for low, moderate, and substantial level of heterogeneity. Subgroup analyses were defined a priori including menopausal status, hormone receptor status, number of outcomes, length of follow-up, geographic location, year of study entry, stage, and treatment. Sources of heterogeneity were explored by meta-regression. Sensitivity analysis was performed to explore the variation between studies by excluding studies based on study methodological quality (high/low risk of bias). As 18 studies included stage 4 patients, these studies were excluded in a sensitivity analysis. All analyses were conducted in R version 4.0.2 using packages ‘meta’, ‘dosresmeta’, and ‘ckbplotr’ [19–21].

## Results

### Search results

**Fig. 1:**
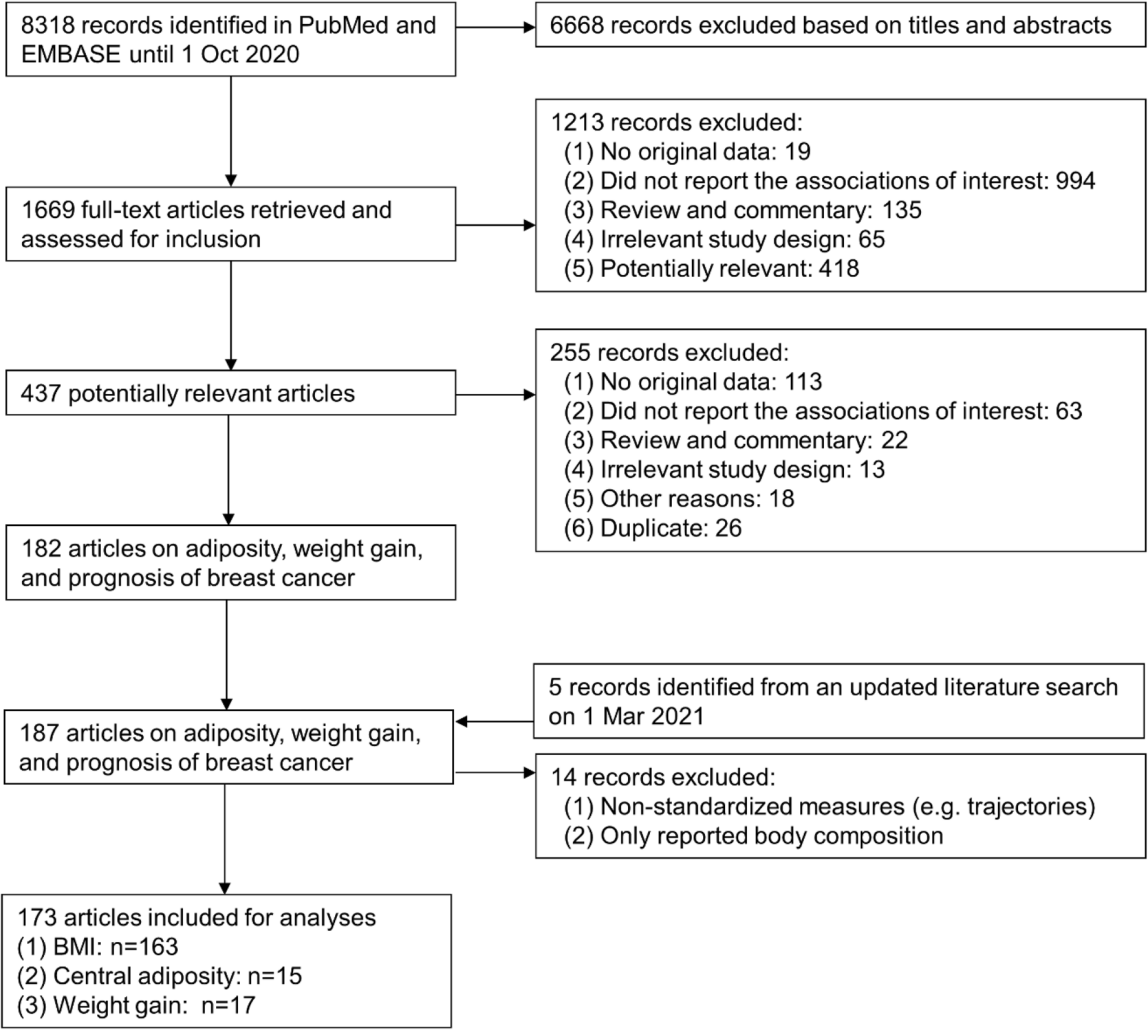
Flow diagram. A flow diagram of studies included in the meta-analysis

A total of 437 potentially relevant articles were identified on adiposity in relation to outcomes among breast cancer patients, of which 255 articles were excluded (Fig. 1). Reasons for exclusions were no original data (113 publications), not reporting the associations of interest (63 publications), review or commentary (22 publications), irrelevant study design (13 publications), duplicate (26 publications), and other reasons (18 publications). After these exclusions, 173 publications from 180 prospective studies with 60,249 deaths (25,751 from breast cancer) in 519,544 breast cancer patients were included in the meta-analyses. No RCTs were identified. Supplementary Table S1 shows the characteristics of included studies.

Several types of studies were included: (1) studies of breast cancer patients ascertained from prospective cohort studies of women free of cancer at baseline; (2) follow-up of breast cancer patients identified using hospital records or from cancer registries; (3) follow-up of breast cancer patients in case–control studies or RCTs. There were 163 publications on BMI, 15 publications on central adiposity [22–36], and 17 publications on weight change [29, 34, 36–50]. Of the 17 studies assessing weight change, 5 studies used post-diagnosis weight minus weight 1–2 years prior to diagnosis [29, 43, 45, 46, 49]; 6 studies used post-diagnosis weight minus weight 1–2 years after diagnosis [34, 37, 40–42, 44], 1 study used weight 5.8 years after diagnosis [38], and 5 studies did not report the exact time post diagnosis [36, 37, 39, 47, 48] (Supplementary Table S1). No studies were identified for male patients.

Mean/median age of breast cancer patients was between 36 and 73 years, and all studies included women. 3.2–67% of patients were obese. The majority of studies (92%) included both post-menopausal and pre-menopausal women, and 13 included either [38, 51–62]. Year of diagnosis ranged between 1961 and 2016, with the majority conducted after 1990. Details of tumor characteristics and stage at diagnosis were reported by 69% of included studies and varied across studies. 18 studies included metastatic cases [31, 35, 36, 63–77] and 3 included carcinoma in situ [66, 78, 79].

The majority of studies were conducted in Europe or North America (64%). There were 2 studies from Australia [80, 81], 9 from Italy [22, 33, 70, 72, 82–86], 8 from France [26, 47, 87–92], 8 from Korea [44, 93–98], 6 from Japan [99–104], 17 from Mainland China [25, 27, 29, 34, 35, 63, 105–114], 2 from Taiwan [115, 116], and 5 international studies [45, 56, 117–119]. The total number of breast cancer patients ranged from 50 to 41,021, and the total number of deaths from 7 to 4468. The median/mean follow-up ranged from 1.2 to 10 years.

### BMI

Table 1 and Supplementary Table S2 summarize the results of the meta-analyses on BMI measured at each time period and each outcome (all-cause mortality, BCSM, recurrence, distant recurrence, DFS, and RFS). Morbid obesity, obesity, overweight, and underweight were associated with risk of all-cause mortality, BCSM, recurrence, DFS and RFS events, while morbid obesity, obesity and overweight were associated with distant recurrence and lower RFS. The evidence was most reliable for BMI <1 year post diagnosis, a measure reported by the majority of studies. The evidence for BMI before diagnosis and BMI  ≥1 year post diagnosis was generally consistent with that for BMI <1 year post diagnosis (Supplementary Figs. S1–S3).

**Table 1:** Meta-analyses of associations of BMI with each outcome

	BMI before diagnosis				BMI <1 year post diagnosis				BMI ≥1 year post diagnosis			
	*N*	RR (95% CI)	*I*^2^ (%)	*p*_het_	*N*	RR (95% CI)	*I*^2^ (%)	*p*_het_	*N*	RR (95% CI)	*I*^2^ (%)	*p*_het_
All-cause mortality												
Under vs normal	9	1.28 (1.02, 1.61)	59.3	0.01	20	1.32 (1.22, 1.43)	16.2	0.25	5	1.50 (1.13, 1.99)	74.7	0.003
Over vs normal	14	1.07 (1.00, 1.15)	20.0	0.24	60	1.13 (1.07, 1.18)	45.8	< 0.001	5	0.98 (0.90, 1.06)	0	0.63
Obese vs normal	17	1.28 (1.19, 1.37)	18.1	0.24	58	1.21 (1.15, 1.27)	45.9	< 0.001	9	1.10 (1.01, 1.19)	0	0.55
Obese vs non-obese	1	1.00 (0.86, 1.16)	–	–	20	1.30 (1.12, 1.51)	79.7	< 0.001	0	–	–	–
Morbidly obese vs normal	1	1.06 (0.62, 1.81)	–	–	10	1.32 (1.03, 1.67)	70.1	< 0.001	3	1.11 (0.96, 1.29)	0	0.42
per 5 units	12	1.10 (1.06, 1.13)	28.4	0.17	22	1.11 (1.05, 1.16)	74.6	< 0.0001	5	1.03 (1.00, 1.07)	0	*0.42*
BCSM												
Under vs normal	6	0.94 (0.81, 1.10)	0	0.53	9	1.35 (1.11, 1.64)	35.2	0.14	2	1.39 (0.81, 2.36)	0	0.81
Over vs normal	10	1.06 (0.98, 1.14)	0	0.71	24	1.12 (1.07, 1.16)	0	0.53	2	1.07 (0.93, 1.24)	0	0.35
Obese vs normal	13	1.17 (1.08, 1.27)	0	0.72	27	1.22 (1.13, 1.32)	37.8	0.03	2	1.49 (0.69, 3.22)	89.2	0.002
Obese vs non-obese	1	1.36 (1.04, 1.78)	–	–	1	1.34 (1.26, 1.42)	–	–	0	–	–	–
Morbidly obese vs normal	1	1.06 (0.74, 1.52)	–	–	2	1.31 (1.10, 1.56)	81.9	0.004	0	–		
per 5 units	9	1.06 (1.02, 1.10)	0	0.65	13	1.11 (1.08, 1.15)	0	0.5	0	–		
Recurrence												
Under vs normal	2	1.10 (0.80, 1.27)	0	0.50	3	1.08 (0.94, 1.24)	0	0.46	1	–		
Over vs normal	5	1.10 (0.97, 1.25)	0	0. 69	15	1.05 (0.98, 1.13)	39.9	0.06	1	–	–	–
Obese vs normal	6	1.21 (0.98, 1.50)	47.7	0.09	18	1.12 (1.06, 1.18)	0	0.83	2	1.40 (1.14, 1.74)	0	0.97
Obese vs non-obese	1	–	–	–	0	–			0	–		
Morbidly obese vs normal	0	–	–	–	1	–	–	–	0	–	–	–
per 5 units	4	1.19 (0.99, 1.42)	77.1	0.005	9	1.07 (1.01, 1.13)	48.6	0.049	0	–		

*BMI* body mass index, *BCSM* breast cancer-specific mortality, *RR* relative risk, *p*_het_ *P* value for heterogeneity between studies

For BMI before diagnosis, compared with normal weight women, the summary RRs of all-cause mortality were 1.28 (1.02–1.61) for underweight women, 1.07 (1.00–1.15) for overweight women, and 1.28 (1.19–1.37) for obese women. The corresponding summary RRs of BCSM were 0.94 (0.81–1.10), 1.06 (0.98–1.14), and 1.17 (1.08–1.27), and of recurrence were 1.10 (0.80–1.27), 1.10 (0.97–1.25), and 1.21 (0.98–1.50). There was low to moderate between-studies heterogeneity (*I*^2^: 0–58%).

For BMI <1 year post diagnosis, compared with normal weight women, the summary RRs of all-cause mortality were 1.32 (1.22–1.43) for underweight women, 1.13 (1.07–1.18) for overweight women, 1.21 (1.15–1.27) for obese women, and 1.32 (1.03–1.67) for morbidly obese women; the summary RRs of all-cause mortality comparing obese and non-obese women were 1.30 (1.12–1.51). The corresponding summary RRs of BCSM for underweight, overweight, and obese women were 1.35 (1.11–1.64), 1.12 (1.07–1.16), and 1.22 (1.13–1.32), respectively, and of recurrence were 1.08 (0.94–1.24), 1.05 (0.98–1.13), and 1.12 (1.06–1.18), respectively. There was moderate to high between-studies heterogeneity.

For BMI ≥1 year post diagnosis, compared with normal weight women, the summary RRs of all-cause mortality were 1.50 (1.13–1.99) for underweight women, 0.98 (0.90–1.06) for overweight women, 1.10 (0.99–1.21) for obese women, and 1.11 (0.96–1.29) for morbidly obese women. The corresponding summary RRs of BCSM were 1.39 (0.81–2.36), 1.07 (0.93–1.24), 1.49 (0.69–3.22) for underweight, overweight, and obese women. There was high between-studies heterogeneity for under vs normal weight of total mortality (*I*^2^ = 74.7%) and obese vs normal weight of BCSM (*I*^2^ = 89.2%). For recurrence, there were data from only one study.

### Central adiposity

For central adiposity, previous studies used different measurements and there was generally large between-study heterogeneity (Table 2). Central adiposity before diagnosis and <1 year post diagnosis were combined because of the general consistency observed for BMI by time of assessment. Central adiposity was associated with all-cause mortality, but there was limited evidence for BCSM.

**Table 2:** Meta-analyses of associations of central adiposity with each outcome

	WC					WHR				HC			
	*N*	RR (95% CI)	*I*^2^ (%)		*p*_het_	*N*	RR (95% CI)	*I*^2^ (%)	*p*_het_	*N*	RR (95% CI)	*I*^2^ (%)	*p*_het_
All-cause mortality													
Middle vs low	4	1.11 (0.88, 1.40)	25.7	0.26		9	1.07 (0.90, 1.28)	57.1	0.02	2	0.99 (0.75, 1.31)	0	0.99
High vs low	9	1.47 (1.19, 1.82)	64.9	0.004		12	1.32 (1.14, 1.53)	49.9	0.02	2	1.30 (1.04, 1.61)	0	0.56
Obese vs non-obese	4	1.76 (1.25, 2.50)	60.7	0.05		2	1.50 (1.11, 2.02)	0	0.74	0	–		
BCSM													
Middle vs low	2	1.11 (0.79, 1.56)	0	0.48		4	1.23 (0.89, 1.68)	53.2	0.09	1	1.14 (0.77, 1.68)	–	–
High vs low	1	1.53 (0.97, 2.42)	–	–		4	1.26 (0.84, 1.88)	67.4	0.03	1	1.50 (1.03, 2.18)	–	–
Obese vs non-obese	2	1.92 (0.77, 4.77)	84.5	0.01		0	–			0	–		

*WC* waist circumference, *WHR* waist-to-hip ratio, *HC* hip circumference, *BCSM* breast cancer-specific mortality, *RR* relative risk, *p*_het_ *P* value for heterogeneity between studies

For WC, the summary RR of all-cause mortality was 1.11 (0.88–1.40) comparing middle vs low categories and was 1.47 (1.19–1.82) comparing high vs low categories; the summary RR comparing obese vs non-obese was 1.76 (1.25–2.50) for all-cause mortality and was 1.92 (0.77–4.77) for BCSM.

For WHR, the summary RR of total mortality was 1.07 (0.90–1.28) comparing middle vs low categories and was 1.32 (1.14–1.53) comparing high vs low categories; the corresponding summary RR of BCSM was 1.23 (0.89–1.68) and 1.26 (0.84–1.88), respectively. For HC, the summary RR of total mortality was 0.99 (0.75–1.31) comparing middle vs low categories and was 1.30 (1.04–1.61) comparing high vs low categories.

### Weight change

Large weight gain was associated with all-cause mortality, BCSM, and recurrence (Table 3). For weight change, compared with no change, the summary RR of all-cause mortality was 1.05 (0.93–1.18) for moderate gain and was 1.28 (1.09–1.50) for large gain; the corresponding summary RR of recurrence was 1.17 (0.99–1.40) and 1.30 (1.10–1.54), respectively. For BCSM, the summary RR was 1.08 (0.94–1.23) comparing moderate gain and no gain and was 1.40 (1.08–1.80) comparing large gain and no gain, with large between-studies heterogeneity for the latter (*I*^2^ = 60.3%).

**Table 3:** Meta-analyses of associations of weight gain with each outcome

	Weight change			
	*N*	RR (95% CI)	*I*^2^ (%)	*p*_het_
All-cause mortality				
Moderate gain	11	1.05 (0.93, 1.18)	38.8	0.09
Large gain	10	1.28 (1.09, 1.50)	58.2	0.01
Any gain	4	1.51 (1.22, 1.87)	0	0.85
BCSM				
Moderate gain	8	1.08 (0.94, 1.23)	6	0.38
Large gain	7	1.40 (1.09, 1.80)	60.3	0.02
Any gain	1	1.73 (1.04, 2.87)	–	–
Recurrence				
Moderate gain	3	1.17 (0.99, 1.39)	8.2	0.34
Large gain	3	1.30 (1.10, 1.54)	0	0.64
Any gain	0	–	–	–

*BCSM* breast cancer-specific mortality, *RR* relative risk, *p*_het_ *P* value for heterogeneity between studies

### Subgroup and sensitivity analyses

**Fig. 2:**
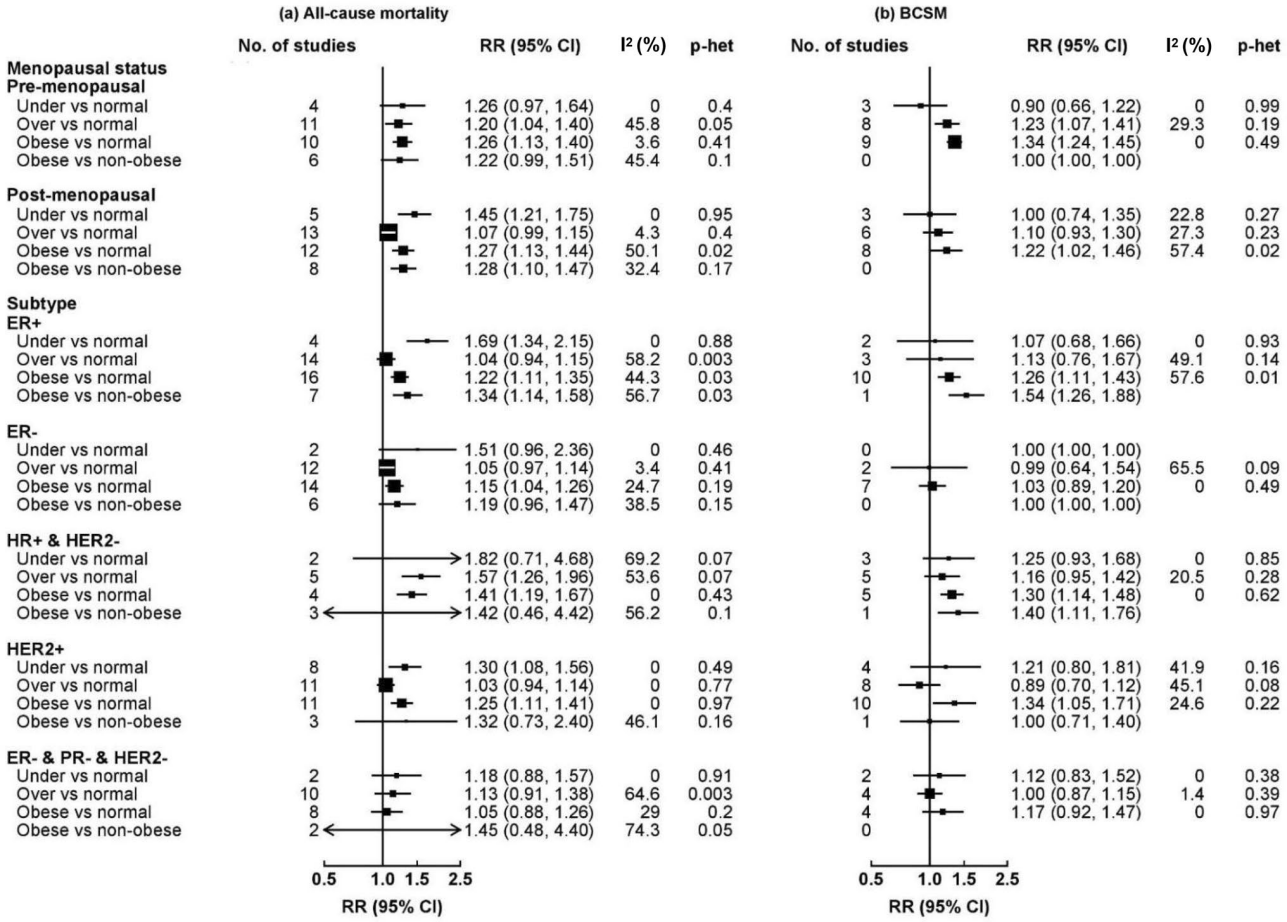
Subgroup analyses of BMI (pre-diagnostic and < 1 year post diagnosis) with overall mortality and BCSM among breast cancer patients. Boxes represent the RRs of **a** all-cause mortality and **b** BCSM associated with obesity, with the area of the box inversely proportional to the variance of the logRR

Subgroup analyses by subtype were only conducted for BMI combining pre-diagnostic BMI and < 1 year post diagnosis (Fig. 2). BMI assessed ≥ 1 year post diagnosis was not included because of the limited number of studies and the possibility of reverse causation. Due to the limited number of studies for central adiposity and weight change, subgroup analyses were only conducted for BMI.

For breast cancer subtypes (Fig. 2), obesity was associated with all-cause mortality among patients with ER+ , ER/PR+ and HER2−, and patients with HER2+ tumors, whereas there were weak associations for patients with ER– and no clear associations for triple-negative breast cancer (TNBC) patients (1.05 [0.88–1.26]). Obesity was associated with BCSM among patients with ER/PR+ and HER2–, and HER2 + tumors, but not among ER– or TNBC patients (ER–: overweight 0.99 [0.64–1.54], obesity 1.03 [0.89–1.20]; TNBC: overweight 1.00 [0.87–1.15], obesity 1.17 [0.92–1.47]).

**Fig. 3:**
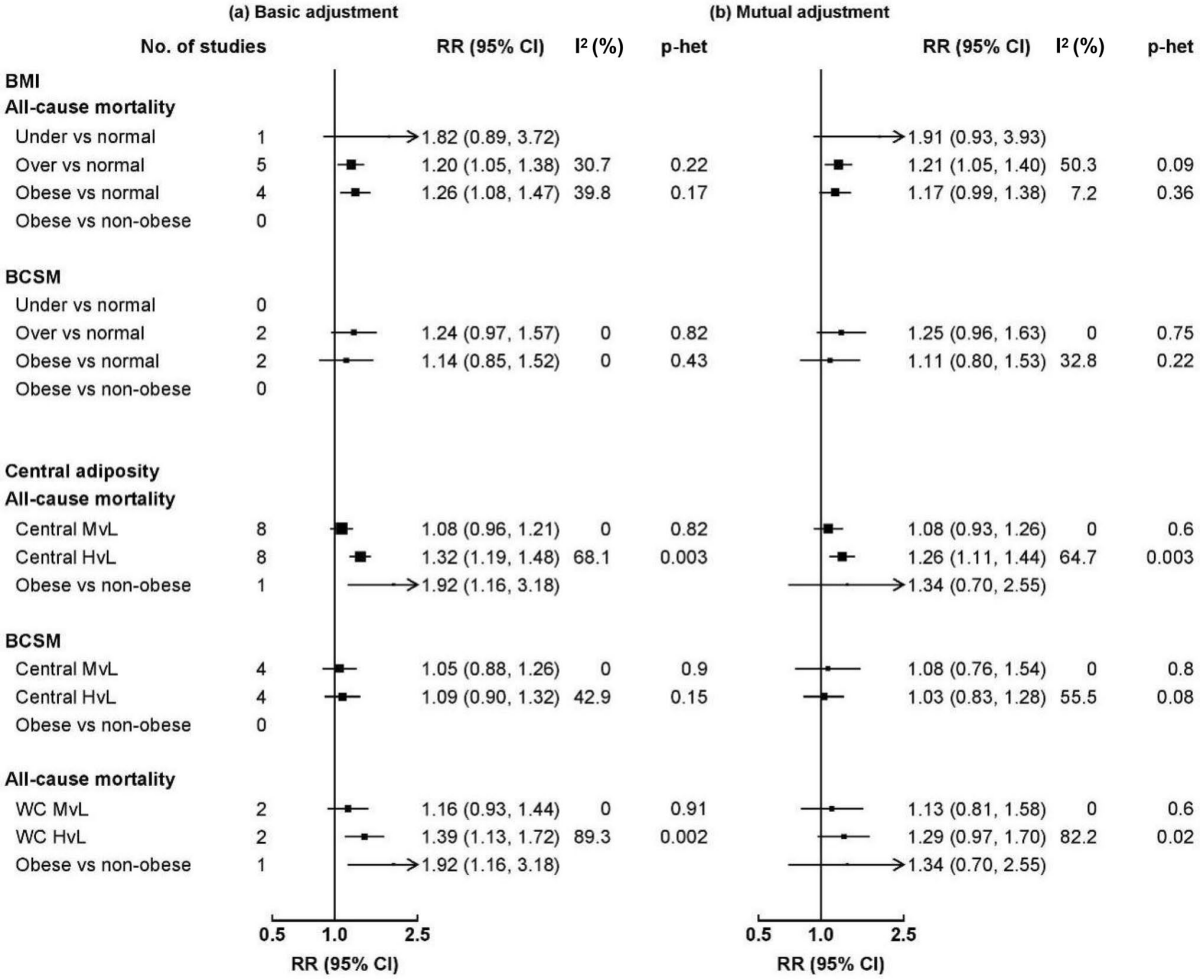
Meta-analysis of BMI and central adiposity in relation to total mortality among breast cancer patients. Boxes represent the hazard ratios (HRs) of all-cause mortality associated with obesity in **a** basic adjustment and **b** mutual adjustment (adjusting BMI for central adiposity and adjusting WC/WHR for BMI), with the area of the box inversely proportional to the variance of the logRR

Due to the limited number of studies reporting BCSM and other outcomes, analyses were only conducted for all-cause mortality comparing BMI and central adiposity with adjustment for each other (Fig. 3). For BMI, the summary RR of all-cause mortality comparing overweight and normal weight was similar with basic adjustment and when further adjusting for WC/WHR, while the summary RR comparing obese and non-obese attenuated slightly when further adjusting for WC/WHR. For central adiposity, the summary RR comparing high and low categories attenuated slightly when further adjusting for BMI, but there was large between-study heterogeneity.

For menopausal status (Fig. 2), underweight, overweight, and obesity were all associated with all-cause mortality, with similar associations among pre- and post-menopausal women. For BCSM, the summary RR tended to be higher in pre-menopausal than post-menopausal women (over vs normal weight: 1.23 [1.07–1.41] and 1.10 [0.93–1.30]; obese vs normal weight: 1.34 [1.24–1.45] and 1.22 [1.02–1.46]), but the differences were non-significant.

For study-level characteristics, meta-regression showed no evidence of significant differences except for region and prevalence of obesity (Supplementary Table S3). For region, the summary RRs of total mortality associated with overweight and obesity were stronger in East Asia than Europe/North America (overweight: 1.24 [1.12–1.36] and 1.07 [1.02–1.12]; obesity: 1.58 [1.38–1.81] and 1.17 [1.12–1.23]). For prevalence of obesity, the summary RR of total mortality associated with obesity was lower in studies with higher prevalence of obesity.

Sensitivity analyses excluding studies which include patients with metastatic breast cancer, or studies with high or unclear risk of bias based on the NOS score yielded similar results compared with the main analyses (Supplementary Table S4).

### Small study effects and publication bias

Asymmetry was detected in the funnel plots for BMI of the following categories and outcomes: (1) pre-diagnosis BMI and BCSM: overweight vs normal weight (Egger test *p* value 0.03) and obese vs normal weight (Egger test *p* value 0.02); (2) BMI <1 year post diagnosis and BCSM: obese vs normal weight (Egger test *p* value 0.002); (3) BMI <1 year post diagnosis and recurrence: overweight vs normal weight (Egger test *p* value 0.04); (4) BMI <1 year post diagnosis and DFS: overweight vs normal weight (Egger test *p* value 0.0005). Funnel plots are shown only for BMI <1 year post diagnosis with all-cause mortality and BCSM (Supplementary Fig. S4).

## Discussion

The present systematic review and meta-analysis showed that general adiposity levels were associated with all-cause mortality, BCSM, any recurrence, and distant recurrence among breast cancer patients. General obesity was associated with higher risk of all-cause mortality and BCSM in patients with ER+ , ER+ and HER2–, and HER2+ tumors, but not in patients with triple-negative tumors. Central obesity was associated with higher risk of all-cause mortality, while large (> 10%) weight gain was associated with higher risk of all-cause mortality, BCSM, and recurrence. The positive associations of general obesity with all-cause mortality and BCSM persisted when additionally adjusted for central adiposity, while the converse was true for central adiposity, suggesting that adiposity overall rather than central adiposity may have a more important role in outcomes.

Our findings of higher risks of all-cause mortality, BCSM, and recurrence comparing underweight to normal weight were consistent with previous meta-analyses [4, 5], and may be related to the presence of comorbid conditions in underweight women. For menopausal status, we observed similar associations of obesity with all-cause mortality and BCSM, consistent with previous meta-analyses [4, 5]. Although several studies have reported inverse associations between obesity and development of breast cancer in pre-menopausal women [120], we found that obesity was associated with higher risks of all-cause mortality and BCSM among both pre- and post-menopausal women with breast cancer.

Findings of this meta-analysis are largely consistent with previous meta-analyses reporting that higher BMI is consistently associated with higher all-cause mortality and BCSM, regardless of when BMI is ascertained. A meta-analysis involving 213,075 breast cancer patients and 41,477 deaths [4] showed that the RRs of all-cause mortality comparing obese vs normal weight were 1.41 (1.29–1.53) for BMI before diagnosis, 1.23 (1.12–1.33) for BMI <1 year after diagnosis, and 1.21 (1.06–1.38) for BMI ≥1 year12 after diagnosis. The corresponding RRs of BCSM were 1.35 (1.24–1.47), 1.25 (1.10–1.42), and 1.68 (0.90–3.15). Pooled estimates in our meta-analysis were slightly different but generally consistent with this meta-analysis. We included different numbers of individual studies by BMI categories because we excluded comparisons with non-standard reference groups, which resulted in lower between-studies heterogeneity for most comparisons. We extended this meta-analysis by showing that higher BMI is also associated with lower risks of recurrence and distant recurrence.

We found that general obesity was associated with all-cause mortality and BCSM in ER/PR+ HER2–, and HER2+ breast cancer patients, while no clear associations were observed in TNBC patients. In contrast, a recent meta-analysis reported that general obesity was associated with all-cause mortality in ER/PR+ HER2–, HER2 + , and TNBC patients [121]. In that meta-analysis, the HR comparing obese vs non-obese for all-cause mortality was 1.39 (1.20–1.62) among ER/PR+ HER2– and 1.32 (1.13–1.53) among TNBC patients. Although that meta-analysis carefully included studies involving the spectrum of immunohistochemically (IHC) defined BC subtypes, it accepted obesity as defined in each study, resulting in high between-study heterogeneity. Nonetheless, some included studies did not present simultaneously the risk estimates across BC subtypes, so it is difficult to establish reliably whether the difference in risk estimates was due to difference in subtypes or other factors. In this context, the Breast Cancer Association Consortium with 121,435 BC patients and 16,890 deaths showed that the associations between obesity and all-cause mortality did not differ by ER status (*P* value > 0.30) [122]. However, only ER status was assessed and associations in more specific BC subtypes (e.g., TNBC) were not assessed in that study. Despite the relatively small number of included studies, our pooled estimates for BCSM by subtype were consistent with subtype-specific estimates for all-cause mortality.

The observed differences by ER status may be related to the fact that adipose tissue produces excess estradiol, leading to higher estrogen exposure particularly in post-menopausal women or women with suppressed ovarian function [123]. Estrogen may only contribute to the association of obesity with outcomes in ER/PR+ BC but not in TNBC patients [124]. Moreover, a Mendelian randomization study showed that genetically predicted obesity is associated with all-cause mortality in ER+ BC patients and is not associated with all-cause mortality in ER– BC patients [6]. Nonetheless, findings from Mendelian randomization studies should be interpreted cautiously because adiposity is associated with both development of BC and outcomes after BC and genetically determined BMI is likely to reflect lifetime adiposity.

Nonetheless, there are other biologic factors involved, including insulin resistance, hyperinsulinemia, dysglycemia, altered adipokines, and inflammation [123]. These biologic effects are more specific to central obesity and they are potentially relevant across BC subtypes, regardless of endogenous estrogen levels. Future research is warranted to explore the potential mechanisms of biologic mediators to outcomes across BC subtypes.

We found that the association between obesity and all-cause mortality was stronger in East Asians than in Europeans. A previous meta-analysis showed a positive association between obesity and all-cause mortality in Europeans, but a null association in East Asians. However, only two East Asian studies were included. Our meta-analysis included 8 individual studies in East Asia which had low between-studies heterogeneity. The stronger association between obesity and all-cause mortality among BC patients may be explained by the high degree of central adiposity in East Asians at the same BMI level compared with Europeans [125], as well as differences in hormonal or reproductive factors (e.g., age at menarche, age at first birth). Although the exact mechanisms for the stronger association of obesity in East Asians need to be understood, if the observed associations are causal, weight management may yield greater benefit in East Asians.

Our estimates for weight change are largely consistent with a previous meta-analysis [126]. Similarly to that meta-analysis, we were unable to conduct subgroup analyses because of the small number of studies included for each outcome. In addition to general adiposity, we showed that central obesity was associated with all-cause mortality and BCSM among BC patients. Importantly, we showed that the associations of central obesity with all-cause mortality and BCSM persisted when additionally adjusting for BMI. This suggests that the mechanisms linking central obesity and BC outcome may not be confined to the effects of estrogen. As discussed above, insulin resistance, hyperinsulinemia, dysglycemia, adipokines, and inflammation may play a role [123, 124], but more studies are needed to understand the mechanisms.

Strengths of this meta-analysis include the breadth of literature search, the inclusion of various adiposity traits and prognostic outcomes, and inclusion of detailed study characteristics. Key factors associated with BC outcomes, such as age and stage, were adjusted for in the majority of studies. This meta-analysis also included studies across wide geographical regions, and therefore the results are readily generalizable. Our study has limitations. First, the subgroup analyses were limited by the number of individual studies reporting subgroup-specific results, such as menopausal status. As a result, our subgroup analyses were only conducted for BMI, but not for central obesity or weight gain. Second, several studies reporting BC subtypes did not specify the details of hormone receptors and HER2 identification and differences are likely to exist between studies and over time. However, our subtype-specific estimates were broadly consistent with previous meta-analyses restricting to studies that used fluorescence in situ hybridization. Third, not all studies defined BC outcomes according to the STEEP definitions, so we used the BC outcomes as defined by individual studies. Academic societies and organizations are to promote the adherence to STEEP definitions in prognostic research. Lastly, there are other sources of heterogeneity including adjustment for potential confounders (patient, tumor, and treatment characteristics) and differences in treatment over time and between countries.

In conclusion, overweight and obesity are associated with higher risks of all-cause mortality, BCSM, recurrence, and distant recurrence among BC patients. The association of general obesity with all-cause mortality is observed among all patients except TNBC. Central adiposity is associated with higher risk of all-cause mortality, and the association persisted after adjustment for BMI. Weight gain is associated with higher risk of all-cause mortality, BCSM, and recurrence. Maintaining a healthy body weight is likely to be beneficial in lowering risk of mortality and recurrence among BC patients, but more studies are warranted to understand the mechanisms of biologic mediators to outcomes across BC subtypes.

## Supplementary Information

Below is the link to the electronic supplementary material.

Supplementary file1 (DOCX 2681 kb)
