# Supplementary material for: Associations of adiposity and weight change with recurrence and survival in breast cancer patients: a systematic review and meta-analysis
Source: Breast Cancer. 2022 May 17;29(4):575–88. doi: 10.1007/s12282-022-01355-z (PMC9226105; doi:10.1007/s12282-022-01355-z)
Supplement: Supplementary file 1 — Supplementary file1 (DOCX 2681 kb) [file 12282_2022_1355_MOESM1_ESM.docx]

**Supplementary materials**

**Associations of adiposity and weight change with recurrence and survival in breast cancer patients: a systematic review and meta-analysis**

Yuanjie Pang, DPhil^1^, Yuxia Wei, MS^2^, Christiana Kartsonaki, DPhil^3,4^

1. Department of Epidemiology and Biostatistics, School of Public Health, Peking University, 38 Xueyuan Road, Beijing 100191, China

2. Institute of Environmental Medicine, Karolinska Institutet, C6 Institutet för miljömedicin, 171 77 Stockholm

3. Clinical Trial Service Unit & Epidemiological Studies Unit (CTSU), Nuffield Department of Population Health, Big Data Institute Building, Roosevelt Drive, University of Oxford, UK

4. Medical Research Council Population Health Research Unit (MRC PHRU), Nuffield Department of Population Health, University of Oxford, UK

Address for correspondence:

Christiana Kartsonaki

MRC PHRU

NDPH, Big Data Institute Building

University of Oxford

Old Road Campus

Oxford, OX3 7LF, UK

Tel: 44-1865-743644

Fax: 44-1865-743985

20 February 2022

Table of Contents

[Supplementary Table S1. Characteristics of included studies 3](#_Toc94023299)

[Supplementary Table S2. Meta-analyses of BMI and risk of recurrence 26](#_Toc94023301)

[Supplementary Table S3. Meta-analyses of BMI and prognosis outcomes among breast cancer patients by subgroup 27](#_Toc94023302)

[Supplementary Table S4. Meta-analyses of BMI and outcomes among breast cancer patients 31](#_Toc94023303)

[Supplementary Figure S1. Meta-analysis of BMI before diagnosis and all-cause mortality 33](#_Toc94023304)

[Supplementary Figure S2. Meta-analysis of BMI before diagnosis and BCSM 34](#_Toc94023306)

[Supplementary Figure S3. Meta-analysis of BMI before diagnosis and recurrence 35](#_Toc94023307)

[Supplementary Figure S4. Funnel plot 36](#_Toc94023308)

[PROSPERO Protocol 37](#_Toc94023309)

# Supplementary Table S1. Characteristics of included studies

| **Year, First author, country** | **Study design, year of diagnosis** | **Follow-up** | **No. cases** | **No. death** | **Age** | **BMI assessment period** |
| --- | --- | --- | --- | --- | --- | --- |
| 2021, Tan, China | Retrospective survivorship cohort study, 2003-2010 | median 70.7 months | 315 | NA | 37.7% 50+ | BMI <1 year post-diagnosis |
| 2021, Shang, US | Retrospective survivorship cohort study, 2000-2017 | median 6.4 years | 2888 | 387 | mean 54.7 for white, 59.6 for black | BMI <1 year post-diagnosis |
| 2021, Martel, US | Survivorship cohort study based on patients in an RCT, 1999-2007 | median 4.5 years | 8381 | NA | 10.2% 65+ | BMI <1 year post-diagnosis |
| 2021, Kennard, US | Retrospective survivorship cohort study, 2007-2013 | median 5.8 years | 177 | 37 | mean 59.9 | BMI <1 year post-diagnosis |
| 2021, Iwase, US | Retrospective survivorship cohort study, 2007-2015 | median 4.7 years | 198 | NA | median 49 | BMI <1 year post-diagnosis |
| 2020, Rasmy, Saudi Arabia | Retrospective survivorship cohort study, 2010-2013 | median 4.1 years | 80 | 7 | mean 50.8 | BMI <1 year post-diagnosis |
| 2020, Ligorio, | Retrospective survivorship cohort study, 2008-2018 | NA | 505 | NA | NA | BMI <1 year post-diagnosis |
| 2020, Jung, Germany | Retrospective survivorship cohort study, 2002-2015 | median 5.5 years | 2216 | 234 | median 62.9 | baseline (median 3.9 months after diagnosis) and follow-up (median 5.8 years after diagnosis) |
| 2020, Gondo, Japan | Retrospective survivorship cohort study, 2002-2014 | median 4.75 years | 3223 | 189 | mean 54 | BMI <1 year post-diagnosis |
| 2020, Franzoi, Belgium | Retrospective survivorship cohort study, 2016-2019 | median 1.2 years | 50 | NA | mean 61.2 | BMI <1 year post-diagnosis |
| 2020, Engkakul, Thailand | Retrospective survivorship cohort study, 2004-2011 | median 65 months | 400 | 52 | mean 52 | BMI <1 year post-diagnosis |
| 2020, Chen, China | Retrospective survivorship cohort study, 2001-2011 | median 6.75 yeats | 38991 | 616 | median 48 | BMI <1 year post-diagnosis |
| 2020, Cárdenas-Cárdenas, Mexico | Retrospective survivorship cohort study, 2013-2017 | median 2.6 years | 220 | NA | 49-70 | BMI <1 year post-diagnosis |
| 2020, Cantini, Indian | Retrospective survivorship cohort study, 2006-2016 | median 5.52 years | 238 | NA | median 54 | BMI <1 year post-diagnosis |
| 2020, Buono, Italy | Retrospective survivorship cohort study, 2009-2013 | median 7.1 years | 717 | 89 | 36.4 % >55 for non-MetS, 75.1% >55 for MetS | BMI <1 year post-diagnosis |
| 2019, Zhang, China | Retrospective survivorship cohort study, 1971-2011 | 5 years | 512 | NA | mean 51-52 | BMI <1 year post-diagnosis |
| 2019, Wang X, China | Retrospective survivorship cohort study, 2010-2012 | median 4.8 years | 3178 | 345 | median 50 | BMI <1 year post-diagnosis |
| 2019, Wang K, China | Retrospective survivorship cohort study, 2005-2015 | median 2 years | 8394 | NA | mean 50 | BMI <1 year post-diagnosis |
| 2019, Vernaci, Italy | Retrospective survivorship cohort study, 2000-2007 | median 12.7 years | 992 | 272 | median 53 | BMI <1 year post-diagnosis |
| 2019, Tryggvadottir, Sweden | Retrospective survivorship cohort study, 2002-2014 | median 5 years | 1178 | 136 | median 61.4 | body size changes during the first postoperative year |
| 2019, Saleh, France | Retrospective survivorship cohort study, 2008-2016 | median 4.1 years | 12999 | NA | NA | BMI <1 year post-diagnosis |
| 2019, Lee, Korea | Retrospective survivorship cohort study, 2012-2016 | median 53.3 months | 336 | NA | median 51 (30-85) | BMI <1 year post-diagnosis |
| 2019, Kim, Korea | Retrospective survivorship cohort study, 2000-2015 | median 71 months | 5919 | 210 | 50+ 41.3% | BMI <1 year post-diagnosis |
| 2019, Blair, US | Survivorship cohort study based on a population-based case-control study, 1997-2009 | median 94 months | 859 | NA | NA | BMI <1 year post-diagnosis |
| 2019, Arnold, Sweden | Survivorship cohort study based on a population-based cohort study, 1991-2012 | mean 5.2/4.2 yrs | 1241 | 130 | mean 56.8/57.5 | BMI <1 year post-diagnosis, change between 20-50 years |
| 2018, Wisse, Sweden | Retrospective survivorship cohort study, 2002-2016 | median 3.05 yrs | 1640 | 136 | mean 60.9 (24-99) | BMI <1 year post-diagnosis |
| 2018, Sun, China | Retrospective survivorship cohort study, 2004-2012 | median 80 months | 1017 | 115 | 66+ 11% | BMI <1 year post-diagnosis |
| 2018, Song, China | Retrospective survivorship cohort study, 2010-2012 | 58 months | 3178 | NA | NA | BMI <1 year post-diagnosis |
| 2018, Sato, Japan | Retrospective survivorship cohort study, 2004-2013 | 73 months | 1924 | 204 | NA | BMI <1 year post-diagnosis |
| 2018, Mutschler, Germany | Survivorship cohort study based on patients in an RCT, 2001-2005 | median 62.9 months | 1080 | 1816 | median 53 | BMI <1 year post-diagnosis, weight change |
| 2018, Martel, | Retrospective survivorship cohort study | NA | 8381 | unclear | NA | BMI <1 year post-diagnosis, weight change at the 2-year visit |
| 2018, Deluche, France | Retrospective survivorship cohort study, 2007-2016 | median 52.4 months | 119 | unclear | median 56 | BMI <1 year post-diagnosis |
| 2018, Cho, Korea | Retrospective survivorship cohort study, 1996-2013 | NA | 5668 | unclear | median 48 and 52 for BMI<25 and 25+ | BMI <1 year post-diagnosis |
| 2017, Zhang S, China | Retrospective survivorship cohort study, 2004-2015 | NA | 158 | unclear | 50+ 50.2% | BMI <1 year post-diagnosis |
| 2017, Zhang M, China | Retrospective survivorship cohort study, 2002-2006 | 5+ years | 4062 | 326 | mean 53.2 | post-diagnosis BMI (6 months and 60 months) |
| 2017, Veal, US | Retrospective survivorship cohort study, 1997-2006 | 6.7 years | 1925 | 196 | 65+ 21.7% | pre-diagnosis (1-year) and post-diagnosis (3 post visits) |
| 2017, Schvartsman, US | Retrospective survivorship cohort study, 2004-2015 | median 7.1 years | 1998 | unclear | 50+ 51.4% | BMI <1 year post-diagnosis |
| 2017, Liu, Taiwan | Retrospective survivorship cohort study, 2005-2013 | median 7.6 years | 131 | 13 | mean 46.9 | change before surgery from those 6 months post-surgery |
| 2017, Farr, Vienna | Retrospective survivorship cohort study, 2005-2015 | mean 30.1 months | 120 | unclear | mean 52.6 | BMI <1 year post-diagnosis |
| 2017, De La Cruz Ku, | Retrospective survivorship cohort study, 2000-2014 | median 5.1 years | 1415 | 506 | median 49.5 |  |
| 2017, Cespedes Feliciano-2, US | Retrospective survivorship cohort study, 2005-2013 | median 3.4 years | 12590 | unclear | mean 59 | weight change after diagnosis |
| 2017, Cespedes Feliciano-1, US | Retrospective survivorship cohort study, 1996-2013 | median 9 years | 1559 | 980 | mean 58 | BMI <1 year post-diagnosis |
| 2017, Buono, Italy | Retrospective survivorship cohort study, 2009-2013 | median 58.9 years | 841 | 145 | mean 52-66 | BMI <1 year post-diagnosis |
| 2017, Biganzoli, Belgium | Survivorship cohort study based on patients in an RCT | median 15.4 years | 734 | unclear | 46% 50+ | BMI <1 year post-diagnosis |
| 2017, Behrouzi, Iran | Retrospective survivorship cohort study, 2003-2014 | median 61 months | 1021 | 150 | mean 48.3, 50+ 44% | BMI <1 year post-diagnosis |
| 2017, Al Jarroudi, Morocco | Retrospective survivorship cohort study, 2009-2011 | median 42.7 months | 115 | 43 | median 47.1 in normal weight, 45.6 in overweight | BMI after diagnosis |
| 2016, Warren, US | Retrospective survivorship cohort study, 1997-2007 | median 10.8 years | 878 | NA | median 55 | BMI <1 year post-diagnosis |
| 2016, Paul, US | Retrospective survivorship cohort study, 2004-2010 | median 68 months | 74 | NA | NA | BMI <1 year post-diagnosis |
| 2016, Nelson, US | Survivorship cohort study based on a population-based cohort study, 1976-2006 | mean 11.8 years | 9513 | 2212 | mean 59 | BMI 1.4 years post-diagnosis |
| 2016, Nechuta, US | Retrospective survivorship cohort study, 1976-2006 | median 12 years | 6295 | 1427 | mean 59.4 | BMI 2.1 years after diagnosis, pre- to post-diagnosis weight change |
| 2016, McCullough, US | Retrospective survivorship cohort study, 1996-1997 | 15 years | 1308 | 441 | 50+ 71.5% | BMI 3 months post-diagnosis |
| 2016, Leachman, | Retrospective survivorship cohort study, 1978-2015 | median 85 months | 3850 | 314 | median 58 | BMI <1 year post-diagnosis |
| 2016, Kawai, Japan | Retrospective survivorship cohort study, 2004-2006 | median 6.7 years | 20090 | 1418 | mean 57.3 | BMI <1 year post-diagnosis |
| 2016, His, France | Retrospective survivorship cohort study, 1995-2008 | median 9.1 years | 3006 | 286 | mean 61.5 | BMI before diagnosis (19.2 months before) |
| 2016, Gennari, Italy | Survivorship cohort study based on patients in an RCT, 1997-2004 | median 103 months | 959 | 139 | median 52 | BMI <1 year post-diagnosis |
| 2016, El-Sadda, | Retrospective survivorship cohort study, 2011-2013 | NA | 500 | NA | NA | BMI <1 year post-diagnosis |
| 2016, D'Aiuto, Italy | Retrospective survivorship cohort study, 2007-2013 | NA | 86 | NA | mean 38 | BMI <1 year post-diagnosis |
| 2016, Chen, China | Retrospective survivorship cohort study, 2006-2015 | NA | 206 | 38 | median 48.5 | BMI <1 year post-diagnosis |
| 2016, Cecchini, US | Retrospective survivorship cohort study, 1999-2014 | mean 5.9 years | 15538 | 2581 | mean 49.6-54.1 | BMI <1 year post-diagnosis |
| 2016, Bergom, US | Retrospective survivorship cohort study, 1998-2010 | median 73 months | 193 | NA | median 60 | BMI <1 year post-diagnosis |
| 2016, Beebe-Dimmer, US | Survivorship cohort study based on a population-based cohort study, | mean 9.9 years | 4347 | 956 | NA | WC at diagnosis |
| 2016, Bao, China | Retrospective survivorship cohort study, 2002-2006 | median 9.1 years | 518 | 10 | mean 53.4 | BMI at diagnosis (18, 36, 60, and 120 months after diagnosis) |
| 2015, Zhang, China | Retrospective survivorship cohort study, 2009-2012 | median 16 months | 1699 | 17 | 50+ 64.4% | BMI <1 year post-diagnosis |
| 2015, Widschwendter, German | Survivorship cohort study based on patients in an RCT, 2005-2007 | median 65 months | 3754 | 302 | median 53 | BMI <1 year post-diagnosis |
| 2015, Sun, US | Retrospective survivorship cohort study, 1993-2001 | median 13.5 years | 1086 | 435 | mean 51 | BMI <1 year post-diagnosis |
| 2015, Shariff-Marco, US | Retrospective survivorship cohort study, 1995-2008 | mean 7.4 years | 4347 | 895 | 55+ 40% | BMI 1 year prior to diagnosis, weight change 1 year before and the interview |
| 2015, Scholz, Germany | Survivorship cohort study based on patients in an RCT, 2001-2005 | median 105.1 months | 1310 | 225 | 60+ 30.9% | BMI <1 year post-diagnosis |
| 2015, Ohara, Japan | Retrospective survivorship cohort study, 2002-2012 | median 46.1 months | 184 | NA | median 64 | BMI <1 year post-diagnosis |
| 2015, Nechuta, US, UK, China | Retrospective survivorship cohort study | mean 9.5 years | 12240 | 401 | NA | BMI on average 1.8 years after diagnosis |
| 2015, Naito, Japan | Survivorship cohort study based on patients in an RCT, | median 6.4 years |  | NA | median 56 |  |
| 2015, Jeon, Korea | Retrospective survivorship cohort study, 1988-20008 | median 92 months | 41021 | 4468 | mean 48 | BMI <1 year post-diagnosis |
| 2015, Herlevic, | Retrospective survivorship cohort study, 1997-2013 | median 49 months | 523 | 86 | mean 61.3 | BMI <1 year post-diagnosis |
| 2015, Hao, China | Retrospective survivorship cohort study, 2002-2012 | median 44.8 months | 1106 | 154 | median 50 and 53 | BMI <1 year post-diagnosis |
| 2015, Crispo, Italy | Retrospective survivorship cohort study, 2004-2006 | median 5 years | 448 | NA | NA | BMI <1 year post-diagnosis |
| 2015, Copson, UK | Retrospective survivorship cohort study, 2001-2007 | median 5.87 years | 2843 | NA | median 36 | BMI <1 year post-diagnosis |
| 2014, Zhang, US | Retrospective survivorship cohort study | median 6.7 years | 202 | 44 | median 71.7 | BMI <1 year post-diagnosis |
| 2014, Tait, US | Retrospective survivorship cohort study, 2006-2010 | median 53 months | 448 | 154 | median 40.1 | BMI <1 year post-diagnosis |
| 2014, Robinson, Australia | Retrospective survivorship cohort study, 2004-2006 | median 5.6 years | 1199 | 43 | mean 58.4 | BMI <1 year post-diagnosis |
| 2014, McLaughlin, US | Retrospective survivorship cohort study, 1997-2006 | mean 6.7 years | 1925 | NA | 21.7% 65+ | BMI pre- and post-diagnosis and BMI change |
| 2014, Ladoire, France | Survivorship cohort study based on patients in an RCT, 1998-2001 | median 5.9 years | 4996 | 668 | mean 49.9 | BMI <1 year post-diagnosis |
| 2014, Jeon, Korea | Retrospective survivorship cohort study, 2005-2010 | median 60.2 years | 108 | NA | mean 50.2 | BMI <1 year post-diagnosis, weight change |
| 2014, George, US | Survivorship cohort study based on a population-based cohort study, 1995-2010 | 9.5 years | 621 | 107 | mean 57 | WC WHR 30 months post-diagnosis |
| 2014, Cihan, Turkey | Retrospective survivorship cohort study, 2005-2013 | mean 126 months | 456 | 48 | mean 55.6 | BMI <1 year post-diagnosis |
| 2014, Calip, US | Retrospective survivorship cohort study, 1990-2008 | median 6.3 years | 4216 | 929 | median 63 | BMI 1 year before diagnosis |
| 2014, Berrino, Italy | Retrospective survivorship cohort study, 2008-2012 | mean 2.8 years | 2092 | NA | NA | BMI <1 year post-diagnosis |
| 2014, Arce-Salinas, Mexico | Retrospective survivorship cohort study, 2004-2008 | median 28 months | 819 | 203 | mean 49 | BMI <1 year post-diagnosis |
| 2013, Xing, China (Shenyang) | Retrospective survivorship cohort study, 2001-2008 | NA | 1192 | 122 | mean 51 | BMI <1 year post-diagnosis |
| 2013, Xiao, US | Retrospective survivorship cohort study, 2007-2010 | NA | 1368 | NA | NA | BMI <1 year post-diagnosis |
| 2013, Pajares, Spain | Survivorship cohort study based on patients in an RCT, 1996-2008 | median 93.4 months | 5683 | 818 | median 48, 56, 55 for BMI<30, 30-34.9, and 35+ | BMI <1 year post-diagnosis |
| 2013, Minicozzi, Italy | Retrospective survivorship cohort study, 2003-2008 | mean 4.3 years | 1607 | NA | 50+ 74% | BMI <1 year post-diagnosis |
| 2013, Kamineni, US | Retrospective survivorship cohort study, 1988-1993 | 10 years | 485 | 97 | 50+ 89% | BMI <1 year post-diagnosis |
| 2013, Jiralerspong, US | Retrospective survivorship cohort study, 1996-2005 | median 5.4 years | 6342 | 951 | median 53, 50+ 60% | BMI <1 year post-diagnosis |
| 2013, Hou, China | Retrospective survivorship cohort study, 2002-2006 | median 68 months | 5634 | NA | 17.7% >65 | BMI <1 year post-diagnosis |
| 2013, Gnant, Austria | Survivorship cohort study based on patients in an RCT | median 73.2 months | 634 | 94 | median 63-69 | BMI <1 year post-diagnosis |
| 2013, Crozier, US | Survivorship cohort study based on patients in an RCT | median 5.3 years | 3017 | 360 | median 46-51 | BMI <1 year post-diagnosis |
| 2013, Contiero, Italy | Retrospective survivorship cohort study, 1996-2000 | median 9.5 years | 1261 | 317 | mean 57 | BMI <1 year post-diagnosis |
| 2013, Connor, US | Retrospective survivorship cohort study, 1992-1994 | mean 13 years | 577 | 216 | mean 53.2 | BMI <1 year post-diagnosis |
| 2013, Asaga, Japan | Retrospective survivorship cohort study, 2000-2009 | median 49.2 months | 135 | NA | median 54 | BMI <1 year post-diagnosis |
| 2012, Panagopoulou, Greece | Retrospective survivorship cohort study, 1997-2005 | median 5.08 years | 2789 | NA | mean 52 | BMI <1 year post-diagnosis |
| 2012, Lee, Korea | Retrospective survivorship cohort study, 1994-2008 | median 35.4 months | 438 | 49 | median 45, 35+ 89% | BMI <1 year post-diagnosis |
| 2012, Kwan, China, US | Survivorship cohort study based on a population-based cohort study, 1990-2006 | mean 7.8 years | 14948 | 2140 | mean 57.3 | BMI 1-2 years before diagnosis |
| 2012, He, US | Retrospective survivorship cohort study, 1998-2010 | median 47.6 months | 2792 | NA | median 55-56 | BMI <1 year post-diagnosis |
| 2012, Hamelinck, Netherland | Retrospective survivorship cohort study, 1997-2004 | NA | 1955 | NA | NA | BMI <1 year post-diagnosis |
| 2012, Haakinson, US | Retrospective survivorship cohort study, 2000-2008 | median 2.5 years | 1352 | NA | mean 66 | BMI <1 year post-diagnosis |
| 2012, Goodwin, Canada | Retrospective survivorship cohort study, 1989-1996 | NA | 535 | NA | mean 50.3 | BMI 1 year after diagnosis |
| 2012, Ewertz, | Survivorship cohort study based on patients in an RCT, 1998-2003 | median 8.7 years | 4760 | 829 | median 60-62 | BMI <1 year post-diagnosis |
| 2012, Dawood, US | Retrospective survivorship cohort study, 1990-2010 | NA | 2311 | 753 | 50+ 51.3% | BMI <1 year post-diagnosis |
| 2012, Caan, US, China | Survivorship cohort study based on a population-based cohort study, 1990-2006 | mean 8.1 years | 12915 | 1603 | mean 57 | weight change (1yr pre- and 2.1 yrs post-diagnosis) |
| 2012, Bradshaw, US | Retrospective survivorship cohort study, 1996-2005 | median 8.8 years | 1033 | 292 | mean 59 | BMI 1year pre-diagnosis, weight change (1year pre- and 1year post-diagnosis) |
| 2012, Armengol-Alonso, | Retrospective survivorship cohort study, 2004-2011 | median 26 months | 108 | NA | median 46 for BMI<25 and 52 for BMI 25+ | BMI <1 year post-diagnosis |
| 2011, Maskarinec, US | Retrospective survivorship cohort study, 1995-1996 | median 13.2 years | 382 | 115 | mean 59.3 | BMI <1 year post-diagnosis |
| 2011, Majed, France | Retrospective survivorship cohort study, 1981-1990 | NA | 15166 | 1370 | mean 54.1 | BMI <1 year post-diagnosis |
| 2011, Lu, US | Retrospective survivorship cohort study, 1994-1998 | median 8.6 years | 4538 | 1053 | mean 49.7 | BMI 5 years pre-diagnosis |
| 2011, Gondou, | Retrospective survivorship cohort study, 2003-2006 | median 59 months | 1100 | 66 | NA | BMI <1 year post-diagnosis |
| 2011, Buck, Germany | Retrospective survivorship cohort study, 2002-2005 | median 6.1 years | 1140 | 162 | mean 62.8 | BMI <1 year post-diagnosis |
| 2011, Baumgartner, Germany | Retrospective survivorship cohort study, 1984-2006 | mean 7.3 years | 1053 | NA | 27-94 | BMI 1 year after diagnosis |
| 2011, Ademuyiwa, US | Retrospective survivorship cohort study, 1996-2010 | median 37.2 months | 418 | 87 | median 54 | BMI <1 year post-diagnosis |
| 2010, Thivat, France | Retrospective survivorship cohort study, 1976-1989 | median 20.4 years | 111 | 57 | median 54 | relative percent weight variation between baseline and postchemotherapy treatment |
| 2010, Sestak, worldwide | Survivorship cohort study based on patients in an RCT, | median 100 months | 4939 | 986 | 65+ 27% | BMI <1 year post-diagnosis |
| 2010, Oh, Korea | Retrospective survivorship cohort study, 1998-2004 | NA | 1357 | 111 | NA | BMI <1 year post-diagnosis, weight at 2 years prior to diagnosis, weight at age 18 |
| 2010, Lu, US | Retrospective survivorship cohort study, 1994-1998 | median 8.6 years | 4538 | 1053 | NA | BMI <1 year post-diagnosis |
| 2010, Keegan, US | Retrospective survivorship cohort study, 1991-2000 | median 7.8 years | 4153 | 725 | 50+ 41% | BMI 19.2 months after diagnosis |
| 2010, Imkampe, UK | Retrospective survivorship cohort study, 1983-2007 | mean 85 months | 2298 | 453 | mean 59 | BMI <1 year post-diagnosis |
| 2010, Giordano, US | Retrospective survivorship cohort study, 1996-2005 | median 5.4 years | 6106 | 921 | median 52 | BMI <1 year post-diagnosis |
| 2010, Flatt, US | Survivorship cohort study based on a population-based cohort study, 1991-2000 | median 7.3 years | 3088 | 315 | mean 52 | BMI 2 year after diagnosis |
| 2010, de Azambuja, | Retrospective survivorship cohort study, 1998-2001 | median 62.5 months | 2887 | 403 | median 52 in obese and 48 in nonobese | BMI <1 year post-diagnosis |
| 2010, Clough-Gorr, US | Retrospective survivorship cohort study | median 7 years | 400 | NA | mean 73 | BMI <1 year post-diagnosis |
| 2010, Chen, China | Retrospective survivorship cohort study, 2002-2006 | median 46 months | 5042 | 442 | median 54 | BMI 6months post-diagnosis, weight change from pre-diagnosis to 6 and 18 months post-diagnosis |
| 2009, Rosenberg, Sweden | Retrospective survivorship cohort study, 1993-1995 | median 9.5 years | 2640 | 83 | mean 62-64 | BMI <1 year post-diagnosis |
| 2009, Olsson, Sweden | Survivorship cohort study based on patients in an RCT, 1961-1991 | 10 years | 2974 | 1318 | mean 61-65 | BMI <1 year post-diagnosis |
| 2009, Nichols, US | Retrospective survivorship cohort study, 1988-2005 | mean 6.3 years | 3993 | 421 | mean 58 | BMI 1-2 years post-diagnosis, BMI 1-5 yrs pre-diagnosis |
| 2009, Moon, Korea | Retrospective survivorship cohort study, 1982-1996 | NA | 4345 | NA | mean 42-52 | BMI <1 year post-diagnosis |
| 2009, Li, US | Survivorship cohort study based on a population-based case-control study, 1990-2005 | NA | 1091 | NA | 50+ 81% | BMI <1 year post-diagnosis |
| 2009, Chen, Taiwan | Retrospective survivorship cohort study, 1998-2005 | median 36 months | 858 | 67 | median 45, 35+ 89% | BMI <1 year post-diagnosis |
| 2008, Vitolins, | Retrospective survivorship cohort study, 1980-1999 | median 13.7 years | 636 | 341 | median 52 | BMI <1 year post-diagnosis |
| 2008, Sánchez, Spain | Retrospective survivorship cohort study, 1975-2003 | mean 75-89 months | 682 | NA | mean 55-58 | BMI <1 year post-diagnosis |
| 2008, Majed, France | Retrospective survivorship cohort study, 1981-2004 | median 8 years | 14709 | 3693 | 50+ 66% | BMI <1 year post-diagnosis |
| 2008, Litton, US | Retrospective survivorship cohort study, 1990-2004 | median 4.1 years | 1169 | 194 | median 50 | BMI <1 year post-diagnosis |
| 2008, Dawood, US | Survivorship cohort study based on patients in an RCT, 1974-2000 | median 6 years | 602 | 341 | median 46-52 | BMI <1 year post-diagnosis |
| 2008, Dal Maso, | Retrospective survivorship cohort study, 1991-1994 | mean 12.6 years | 1453 | NA | 23-74 | pre-diagnosis BMI |
| 2008, Caan, US | Retrospective survivorship cohort study, 1997-2000 | mean 83.9 months | 2288 | 250 | mean 58.3 | BMI 1-year pre-diagnosis, at study entry, weight change |
| 2008, Barnett, UK | Retrospective survivorship cohort study, 1991-2005 | median 6.8 years | 4560 | 620 | median 52, 50+ 55% | BMI <1 year post-diagnosis |
| 2007, Pierce, US | Retrospective survivorship cohort study, 1991-2005 | mean 8.7 years | 1490 | 135 | mean 50, 60+ 23% | BMI <1 year post-diagnosis |
| 2007, Cleveland, US | Retrospective survivorship cohort study, 1996-2002 | NA | 1491 | 196 | 50+ 73% | weight change 1-year pre-diagnosis |
| 2006, Tao, China | Retrospective survivorship cohort study, 1996-2002 | median 5.1 years | 1455 | 240 | 50+ 37% | BMI <1 year post-diagnosis |
| 2006, Dignam, US | Survivorship cohort study based on patients in an RCT, 1981-1998 | NA | 4077 | 820 | 50+ 42.6% | BMI <1 year post-diagnosis |
| 2006, Abrahamson, US | Retrospective survivorship cohort study, 1990-1992 | NA | 1254 | 290 | 45+ 32% | BMI <1 year post-diagnosis, change from age 20 to diagnosis, or from diagnosis to interview |
| 2005, Whiteman, US | Retrospective survivorship cohort study, 1980-1982 | median 14.6 years | 3924 | 1671 | 50+ 30% | BMI <1 year post-diagnosis |
| 2005, Loi, Australia | Retrospective survivorship cohort study, 1992 | median 5 years | 1101 | 184 | mean 42.7 | BMI <1 year post-diagnosis |
| 2005, Kroenke, US | Survivorship cohort study based on a population-based cohort study, 1976-2000 | median 9 years | 5204 | 860 | mean 56-61 | BMI >1 year after diagnosis |
| 2005, Gonzalez-Angulo, US | Retrospective survivorship cohort study, 1990-2002 | mean 3 years | 452 | NA | <35 | BMI <1 year post-diagnosis |
| 2004, Maehle, Norway | Retrospective survivorship cohort study, 1963-2000 | median 189 months | 1211 | 471 | median 50 | BMI <1 year post-diagnosis |
| 2004, Enger-2, US | Survivorship cohort study based on a population-based case-cohort study, 1983-2000 | median 10.4 years | 717 | 263 | NA | BMI <1 year post-diagnosis |
| 2004, Carmichael, UK | Retrospective survivorship cohort study, 1963-1999 | mean 6 years | 1579 | NA | NA | BMI <1 year post-diagnosis |
| 2004, Berclaz, international | Survivorship cohort study based on patients in an RCT, 1978-1993 | median 14 years | 6370 | NA | median 48-55 | BMI <1 year post-diagnosis |
| 2003, Dignam, US | Survivorship cohort study based on patients in an RCT, 1982-2001 | median 166 months | 3385 | 983 | 50+ 69% | BMI <1 year post-diagnosis |
| 2001, Marret, France | Retrospective survivorship cohort study, 1976-1988 | median 82 months | 605 | 96 | mean 53 | BMI <1 year post-diagnosis |
| 2001, Daling, US | Retrospective survivorship cohort study, 1983-1992 | NA | 1177 | 317 | <45 | BMI <1 year post-diagnosis |
| 2000, Kumar, US | Retrospective survivorship cohort study | 10 years | 166 | 83 | 60+ 38% | BMI <1 year post-diagnosis |
| 2000, Chang, US | Retrospective survivorship cohort study, 1974-1993 | median 100 months | 177 | 101 | 50+ 52% | BMI <1 year post-diagnosis |
| 1999, Menon, UK | Survivorship cohort study based on patients in an RCT, ~1997 | median 6 years | 403 | 162 | NA | BMI <1 year post-diagnosis |
| 1998, Hebert, US | Retrospective survivorship cohort study, 1982-1984 | NA | 472 | 87 | mean 52.2 | BMI <1 year post-diagnosis |
| 1998, Habel, US | Retrospective survivorship cohort study, 1980-1992 | median 62 month | 480 | 46 | 55+ 50.6% | BMI <1 year post-diagnosis |
| 1997, Newman, Canada | Retrospective survivorship cohort study, 1978-1989 | mean 4.4 years | 1169 | 295 | median 56 | BMI <1 year post-diagnosis |
| 1995, den Tonkelaar, Netherland | Retrospective survivorship cohort study, 1974-1990 | mean 9.1 years | 241 | 63 | NA | BMI <1 year post-diagnosis |
| 1994, Katoh, US | Retrospective survivorship cohort study, 1977-1985 | min 5 years | 301 | 122 | median 72 | BMI <1 year post-diagnosis |
| 1994, Jain, Canada | Retrospective survivorship cohort study, 1981-1992 | mean 5.2 years | 1033 |  | mean 52.2 | BMI <1 year post-diagnosis |
| 1994, Holmberg, Norway and Sweden | Retrospective survivorship cohort study, 1984-1985 | max 5 years | 318 | 94 | <45 | BMI <1 year post-diagnosis |
| 1994, Bastarrachea, US | Retrospective survivorship cohort study, 1974-1982 | median 10.7 years | 735 | 349 | NA | BMI <1 year post-diagnosis |
| 1992, Senie, US | Retrospective survivorship cohort study | 10 years | 923 | 307 | mean 55.5 | BMI <1 year post-diagnosis |
| 1991, Ewertz, Denmark | Retrospective survivorship cohort study, 1983-1984 | NA | 1744 | 805 | 50+ 61% | BMI 1-year post-diagnosis |
| 1990, Mason, New Zealand | Retrospective survivorship cohort study, 1981-1986 | NA | 1770 | 586 | NA |  |
| 1990, Camoriano, US | Retrospective survivorship cohort study | mean 6.6 years | 646 | NA | 20-75 | BMI 1-year post-diagnosis |

**Supplementary Table S1. Continued**

| **Year, First author, country** | **Post-menopausal, %** | **Chemo therapy, %** | **ER+, %** | **PR+, %** | **Stage III-IV, %** | **NOS** |
| --- | --- | --- | --- | --- | --- | --- |
| 2021, Tan, China |  |  | 64.10% | 64.80% |  | 8 |
| 2021, Shang, US |  | 44.1% in white, 47% in black | 80.8% in white, 69.7% in black | 68.9% in white, 57% in black | 11.4% in white, 13.4% in black | 8 |
| 2021, Martel, US | 56.6% | 100% |  |  |  | low |
| 2021, Kennard, US |  | chemo 75% | 0 | 0 |  | 9 |
| 2021, Iwase, US | 43% | 100% | 74.7% | 58.1% | 100% | 6 |
| 2020, Rasmy, Saudi Arabia |  |  | 83.8% | 63.8% |  | 5 |
| 2020, Ligorio, |  |  |  |  |  | unclear |
| 2020, Jung, Germany | 100% |  | 80% | 65% |  | 9 |
| 2020, Gondo, Japan | 53.7% |  | 83.2% | 72.3% |  | 7 |
| 2020, Franzoi, Belgium | 94% |  |  |  |  | 7 |
| 2020, Engkakul, Thailand | 49.8% |  | 77.0% | 77.0% | 38.0% | 8 |
| 2020, Chen, China | 3.9% |  | 58.1% | 61.3% | 27.9% | 5 |
| 2020, Cárdenas-Cárdenas, Mexico | 100% |  |  |  |  | 6 |
| 2020, Cantini, Indian | 58.4% |  |  |  | 16% | 6 |
| 2020, Buono, Italy | 50.2% |  |  |  |  | 8 |
| 2019, Zhang, China |  |  | 56% | 58% |  | 9 |
| 2019, Wang X, China |  |  | 71% | 64% |  | 5 |
| 2019, Wang K, China | 46.8% |  |  |  |  | 9 |
| 2019, Vernaci, Italy | 65% |  |  |  | 12% | 9 |
| 2019, Tryggvadottir, Sweden | 80.9% |  | 89% | 72% |  | 9 |
| 2019, Saleh, France |  |  |  |  |  | unclear |
| 2019, Lee, Korea | 57.4% |  | 74% | 62% | 10.4% | 7 |
| 2019, Kim, Korea | 41.6% |  |  |  | 13.1% | 6 |
| 2019, Blair, US | 77.5% | 44.40% |  |  | 15.5% | 9 |
| 2019, Arnold, Sweden | 100% |  |  |  | 3.6% | 8 |
| 2018, Wisse, Sweden | 80.2% | 27.60% | 89% | 71% |  | 8 |
| 2018, Sun, China | 40.9% | 50.70% | 61% | 63% |  | 9 |
| 2018, Song, China |  |  |  |  |  | unclear |
| 2018, Sato, Japan | 67% |  |  |  |  | unclear |
| 2018, Mutschler, Germany | 59% |  | 76% | 76% | 15.4% | 9 |
| 2018, Martel, |  |  |  |  |  | unclear |
| 2018, Deluche, France | 60% | 54% | 74% | 55% |  | 6 |
| 2018, Cho, Korea |  | 69.2% | 72% | 66% | 5% | 7 |
| 2017, Zhang S, China | 50.2% |  | 62% | 58% | 7% | 6 |
| 2017, Zhang M, China | 50.1% | 92.3% | 66% | 59% | 6.5% | 9 |
| 2017, Veal, US |  |  |  |  |  | 7 |
| 2017, Schvartsman, US | 57.4% |  |  |  |  | 7 |
| 2017, Liu, Taiwan | 34.4% | 83.2% | 70% | 55% | 19.10% | 9 |
| 2017, Farr, Vienna | 61.7% |  | 68% |  |  | 7/5/5 for different outcomes |
| 2017, De La Cruz Ku, | 55% |  |  |  | 46% | unclear |
| 2017, Cespedes Feliciano-2, US |  | 46% |  |  | 10% | 9 |
| 2017, Cespedes Feliciano-1, US | 67% | 54% |  |  | 7% | 8 |
| 2017, Buono, Italy | 60.9% | 100% |  |  | 28.7% | 6 |
| 2017, Biganzoli, Belgium | 42% | 100% | 74% | 68% |  | low |
| 2017, Behrouzi, Iran |  | 100% | 68% | 61% | 34.8% | 9 |
| 2017, Al Jarroudi, Morocco | 26.10% |  | 0 | 0 |  | 7 |
| 2016, Warren, US | 68% | 44.2% | 79% |  |  | 8 |
| 2016, Paul, US |  |  |  |  |  | unclear |
| 2016, Nelson, US | 72.6% |  |  |  |  | 8 |
| 2016, Nechuta, US | 72.9% | 46.2% | 100% | 82% | 11.1% | 8 |
| 2016, McCullough, US | 68.7% | 39.9% | 75% | 64% |  | 8 |
| 2016, Leachman, | 70% |  | 72% |  |  | unclear |
| 2016, Kawai, Japan | 62.6% | 47.1% |  |  | 9.7% | 8 |
| 2016, His, France | 90.8% |  | 83% | 65% | 6.4% | 8 |
| 2016, Gennari, Italy | 53% |  | 62% |  |  | low |
| 2016, El-Sadda, |  |  |  |  |  | unclear |
| 2016, D'Aiuto, Italy |  |  |  |  | 67.4% | 5 |
| 2016, Chen, China | 45% |  |  |  | 8.7% | 7 |
| 2016, Cecchini, US |  | 100% |  |  |  | 9 |
| 2016, Bergom, US | 68% | 33% | 77% | 77% |  | 7/9/7 for different outcomes |
| 2016, Beebe-Dimmer, US |  |  |  |  |  | unclear |
| 2016, Bao, China | 53% | 94% |  |  | 10% | 9 |
| 2015, Zhang, China | 64.1% |  | 65.4% | 43% | 34.4% | unclear |
| 2015, Widschwendter, German | 58% |  | 70% |  | 6.7% | low |
| 2015, Sun, US | 58% |  |  |  |  | 7 |
| 2015, Shariff-Marco, US | 60% | 55% | 69% | 69% | 9% | 8 |
| 2015, Scholz, Germany | 61.8% | 100% | 76% |  | 16.2% | low |
| 2015, Ohara, Japan | 100% | 28.8% | 100% |  | 3% | 6 |
| 2015, Nechuta, US, UK, China |  |  |  |  |  | unclear |
| 2015, Naito, Japan |  |  | 71% |  |  | unclear |
| 2015, Jeon, Korea |  | 70.1% |  |  |  | 8 |
| 2015, Herlevic, |  | 17.6% | 50% | 44% | 15.3% | 9 |
| 2015, Hao, China | 49% | 94% |  |  | 57% | 9 |
| 2015, Crispo, Italy | 68.3% |  | 72% | 71% | 7.4% | 8 |
| 2015, Copson, UK | 0.30% | 91.5% | 66% | 56% |  | 9 |
| 2014, Zhang, US |  | 32% | 85% |  | 11% | unclear |
| 2014, Tait, US | 57.4% | 88% |  |  | 24.1% | 6 |
| 2014, Robinson, Australia |  | 41.3% |  |  |  | 8 |
| 2014, McLaughlin, US | 60% | 33.1% |  |  |  | 8 |
| 2014, Ladoire, France | 43.6% | 100% | 76% | 65% |  | low |
| 2014, Jeon, Korea | 63% |  | 55% | 47% |  | 7 |
| 2014, George, US |  | 13% |  |  |  | 8 |
| 2014, Cihan, Turkey | 56% | 95% | 59% | 54% | 41% | 7 |
| 2014, Calip, US | 73% | 33% | 78% | 70% |  | 8 |
| 2014, Berrino, Italy | 45% |  | 85% | 77% |  | 7 |
| 2014, Arce-Salinas, Mexico | 45% |  | 44% | 55% | 69% | 7 |
| 2013, Xing, China (Shenyang) | 39% | 88% | 55% | 58% |  | 9 |
| 2013, Xiao, US |  | 41.3% | 76.5% | 62% |  | 9 |
| 2013, Pajares, Spain | 46.8% |  | 67% | 60% |  | 8 |
| 2013, Minicozzi, Italy |  | 55% | 62% | 62% | 6% | 8 |
| 2013, Kamineni, US |  | 18% | 73% | 64% |  | 7 |
| 2013, Jiralerspong, US |  | 56% | 77% | 77% |  | 7 |
| 2013, Hou, China | 56.4% | 100% | 65.4% | 69.3% | 13% | 8 |
| 2013, Gnant, Austria | 100% |  | 97% | 88% |  | low |
| 2013, Crozier, US | 47% |  | 54% | 54% |  | low |
| 2013, Contiero, Italy | 65.2% |  | 76.5% | 66.3% | 17.1% | 9 |
| 2013, Connor, US | 58.8% | 42.8% | 61.7% |  |  | 7 |
| 2013, Asaga, Japan | 57.8% | 100% | 0 | 0 |  | 6 |
| 2012, Panagopoulou, Greece | 53% |  | 76% | 76% |  | low |
| 2012, Lee, Korea |  |  | 48% | 33% | 76.6% | 8 |
| 2012, Kwan, China, US | 65.1% | 65.3% | 74% | 65% | 13.1% | 8 |
| 2012, He, US | 40.2% |  |  |  | 33.1% | 9 |
| 2012, Hamelinck, Netherland |  |  |  |  |  | unclear |
| 2012, Haakinson, US |  |  |  |  | 3% | 6 |
| 2012, Goodwin, Canada |  | 39.8% | 67.7% | 61.7% |  | 6 |
| 2012, Ewertz, | 100% | 0 | 71.7% | 64.1% |  | 9 |
| 2012, Dawood, US | 57.50% |  |  |  | 22.8% | 9 |
| 2012, Caan, US, China | 64.6% | 65.9% | 75% | 65% | 12.6% | 8 |
| 2012, Bradshaw, US | 68% | 41% | 74% | 64% |  | 9 |
| 2012, Armengol-Alonso, |  |  |  |  |  | unclear |
| 2011, Maskarinec, US |  |  | 67% | 55% |  | 8 |
| 2011, Majed, France | 47% |  | 50% | 49% |  | 7 |
| 2011, Lu, US | 42% |  | 59% |  |  | 9 |
| 2011, Gondou, |  |  |  |  |  | unclear |
| 2011, Buck, Germany | 100% | 66% |  |  |  | 9 |
| 2011, Baumgartner, Germany |  | 32.2% |  |  |  | unclear |
| 2011, Ademuyiwa, US |  |  |  |  |  | 8 |
| 2010, Thivat, France | 55% | 100% | 42% | 35% |  | 7 |
| 2010, Sestak, worldwide | 100% | 100% |  |  |  | low |
| 2010, Oh, Korea | 31% |  |  |  |  | unclear |
| 2010, Lu, US |  |  |  |  |  | unclear |
| 2010, Keegan, US |  | 49% | 65% | 63% |  | 7 |
| 2010, Imkampe, UK |  | 54% |  | 43% |  | 7 |
| 2010, Giordano, US |  |  | 77% | 77% | 7% | unclear |
| 2010, Flatt, US |  |  | 74% |  | 15.9% | 7 |
| 2010, de Azambuja, | 40% |  | 76% | 76% |  | low |
| 2010, Clough-Gorr, US |  | 22% |  |  |  | 7 |
| 2010, Chen, China | 51% | 91% | 50% | 50% | 10% | 8 |
| 2009, Rosenberg, Sweden | 100% | 0.01% | 565 | 47% |  | 8 |
| 2009, Olsson, Sweden | 14.5% |  |  |  |  | low |
| 2009, Nichols, US | 72% |  |  |  |  | 9 |
| 2009, Moon, Korea |  | 23% | 24% | 24% |  | 8 |
| 2009, Li, US |  | 26% |  |  |  | 9 |
| 2009, Chen, Taiwan | 20% | 74% | 44% | 42% | 11% | 8 |
| 2008, Vitolins, | 59% |  | 62% | 49% |  | 7 |
| 2008, Sánchez, Spain |  | 28% | 55% |  |  | 7 |
| 2008, Majed, France | 55% | 31% | 51% | 50% | 13% | 8 |
| 2008, Litton, US | 51.3% |  | 60.1% | 51.2% | 32.9% | 9 |
| 2008, Dawood, US | 45% | 100% | 40% | 40% | 83% | 7 |
| 2008, Dal Maso, |  |  |  |  |  | 5 |
| 2008, Caan, US | 63.8% | 57.4% | 82.8% | 70.9% | 3.1% | 8 |
| 2008, Barnett, UK |  |  | 45% |  | 4% | 7 |
| 2007, Pierce, US |  | 69% | 73% | 67% | 16% | 8 |
| 2007, Cleveland, US | 68% |  | 73% | 64% |  | 8 |
| 2006, Tao, China |  | 94% | 44% | 44% | 11% | 8 |
| 2006, Dignam, US | 45.5% |  |  |  |  | low |
| 2006, Abrahamson, US | 22% |  | 56% |  |  | 8 |
| 2005, Whiteman, US | 35% |  |  |  |  | 9 |
| 2005, Loi, Australia | 26% | 62% | 61% | 65% |  | 8 |
| 2005, Kroenke, US |  | 32% | 83% |  |  | 8 |
| 2005, Gonzalez-Angulo, US | 6% |  | 45.4% | 40.5% |  | 4 |
| 2004, Maehle, Norway |  |  |  |  |  | 8 |
| 2004, Enger-2, US | 0% |  |  |  |  | 9 |
| 2004, Carmichael, UK | 100% |  |  |  |  | 5 |
| 2004, Berclaz, international | 45% |  | 57% | 48% |  | high |
| 2003, Dignam, US | 69% |  | 100% |  |  | low |
| 2001, Marret, France | 55% |  |  |  |  | 7 |
| 2001, Daling, US |  |  |  |  |  | 9 |
| 2000, Kumar, US | 83% |  |  |  | 7.2% | 7 |
| 2000, Chang, US | 53% |  |  |  |  | 7 |
| 1999, Menon, UK |  |  |  |  |  | 5 |
| 1998, Hebert, US | 53% |  | 57.1% |  | 19.7% | 9 |
| 1998, Habel, US |  |  |  |  |  | 7 |
| 1997, Newman, Canada |  |  |  |  | 6.3% | 9 |
| 1995, den Tonkelaar, Netherland |  |  |  |  |  | 6 |
| 1994, Katoh, US | 100% |  | 78% | 56% | 16% | 7 |
| 1994, Jain, Canada |  |  |  |  |  | 7 |
| 1994, Holmberg, Norway and Sweden | 0% |  |  |  |  | 8 |
| 1994, Bastarrachea, US | 52% |  |  |  | 31% | 6 |
| 1992, Senie, US | 51% |  |  |  |  | 9 |
| 1991, Ewertz, Denmark |  |  |  |  |  | 9 |
| 1990, Mason, New Zealand |  |  |  |  |  | 4 |
| 1990, Camoriano, US | 48.9% |  |  |  |  | 6 |

# Supplementary Table S2. Meta-analyses of BMI and risk of recurrence

|  |  |  |  |  |  |  |  |  |  |
| --- | --- | --- | --- | --- | --- | --- | --- | --- | --- |
|  | **BMI before diagnosis** | | | |  | **BMI <1 year post-diagnosis** | | | |
|  | **N** | **RR (95% CI)** | ***I*^2^ (%)** | ***p*_het_** |  | **N** | **RR (95% CI)** | ***I*^2^ (%)** | ***p*_het_** |
| **Distant metastasis** |  |  |  |  |  |  |  |  |  |
| Under vs normal | 1 | — |  |  |  | 2 | 1.43 (0.86, 2.39) | 41.7 | 0.19 |
| Over vs normal | 1 | — |  |  |  | 8 | 1.15 (1.05, 1.26) | 40.1 | 0.11 |
| Obese vs normal | 1 | — |  |  |  | 9 | 1.19 (1.11, 1.28) | 13.5 | 0.32 |
| Obese vs non-obese | 0 | — |  |  |  | 2 | 1.48 (1.07, 2.03) | 0 | 0.37 |
| Morbidly obese vs normal | 0 | — |  |  |  | 1 | — |  |  |
| **DFS** |  |  |  |  |  |  |  |  |  |
| Under vs normal | 1 |  |  |  |  | 6 | 1.66 (1.22, 2.27) | 55.3 | 0.05 |
| Over vs normal | 2 | 1.18 (0.99, 1.40) | 0 | 0.84 |  | 32 | 1.17 (1.10, 1.24) | 44.8 | 0.004 |
| Obese vs normal | 2 | 1.29 (0.69, 2.38) | 71.1 | 0.06 |  | 25 | 1.17 (1.10, 1.24) | 36.6 | 0.04 |
| Obese vs non-obese | 0 | — |  |  |  | 9 | 1.20 (1.09, 1.32) | 25.0 | 0.22 |
| Morbidly obese vs normal | 0 | — |  |  |  | 2 | 2.58 (1.73, 3.83) | 0 | 0.70 |
| **RFS** |  |  |  |  |  |  |  |  |  |
| Under vs normal | 0 | — |  |  |  | 3 | 2.77 (1.14, 6.74) | 76.0 | 0.02 |
| Over vs normal | 0 | — |  |  |  | 9 | 1.11 (1.01, 1.23) | 18.3 | 0.28 |
| Obese vs normal | 0 | — |  |  |  | 7 | 1.14 (1.01, 1.29) | 37.2 | 0.14 |
| Obese vs non-obese | 0 | — |  |  |  | 5 | 1.37 (1.16, 1.62) | 38.3 | 0.17 |
| Morbidly obese vs normal | 0 | — |  |  |  | 1 | — |  |  |
|  |  |  |  |  |  |  |  |  |  |

Abbreviations: BMI, body mass index; DFS, disease free survival; RFS, recurrence free survival; RR, relative risk.

*p*_het_: *p*-value for heterogeneity between studies

# Supplementary Table S3. Meta-analyses of BMI and prognosis outcomes among breast cancer patients by subgroup

|  |  | | **All-cause mortality** | | | |  |  | **BCSM** |  |  |  |
| --- | --- | --- | --- | --- | --- | --- | --- | --- | --- | --- | --- | --- |
|  | **N** | **RR (95% CI)** | | **I^2^ (%)** | **ph1** | **ph2** |  | **N** | **RR (95% CI)** | **I^2^ (%)** | **ph1** | **ph2** |
| **Region** |  |  | |  |  |  |  |  |  |  |  |  |
| **Under vs normal** |  |  | |  |  |  |  |  |  |  |  |  |
| East Asia | 10 | 1.42 (1.24, 1.64) | | 0 | 0.75 | 0.46 |  | 1 | 1.16 (0.90, 1.50) |  |  |  |
| Europe/North America | 18 | 1.35 (1.18, 1.54) | | 62.5 | 0.0002 |  |  | 14 | 1.16 (0.95, 1.42) | 44.5 | 0.04 |  |
| Other | 2 | 1.59 (1.26, 2.01) | | 0 | 0.98 |  |  | 1 | 1.33 (0.92, 1.92) |  |  |  |
| **Over vs normal** |  |  | |  |  |  |  |  |  |  |  |  |
| East Asia | 20 | 1.24 (1.12, 1.36) | | 35.1 | 0.06 | 0.01 |  | 4 | 1.10 (0.90, 1.34) | 14.3 | 0.32 | 0.95 |
| Europe/North America | 49 | 1.07 (1.02, 1.12) | | 40.2 | 0.002 |  |  | 27 | 1.11 (1.06, 1.16) | 0 | 0.77 |  |
| Other | 4 | 1.01 (0.89, 1.15) | | 50.8 | 0.11 |  |  | 1 | 1.04 (0.92, 1.18) |  |  |  |
| **Obese vs normal** |  |  | |  |  |  |  |  |  |  |  |  |
| East Asia | 9 | 1.58 (1.38, 1.81) | | 0 | 0.76 | 0.0002 |  | 2 | 1.73 (1.01, 2.96) | 42.6 | 0.19 | 0.21 |
| Europe/North America | 46 | 1.17 (1.12, 1.23) | | 30.2 | 0.03 |  |  | 28 | 1.22 (1.14, 1.31) | 26.7 | 0.10 |  |
| Other | 3 | 1.18 (1.06, 1.33) | | 39.4 | 0.19 |  |  | 1 | 1.10 (0.95, 1.28) |  |  |  |
| **Year** |  |  | |  |  |  |  |  |  |  |  |  |
| **Under vs normal** |  |  | |  |  |  |  |  |  |  |  |  |
| <1990 | 8 | 1.46 (1.13, 1.90) | | 77.8 | <0.0001 | 0.78 |  | 9 | 1.23 (0.95, 1.61) | 47.8 | 0.05 | 0.26 |
| 1990-2000 | 10 | 1.33 (1.10, 1.62) | | 49.6 | 0.04 |  |  | 5 | 0.99 (0.82, 1.20) | 0 | 0.47 |  |
| 2000- | 12 | 1.33 (1.22, 1.45) | | 0 | 0.86 |  |  | 2 | 1.60 (0.73, 3.47) | 73.4 | 0.05 |  |
| **Over vs normal** |  |  | |  |  |  |  |  |  |  |  |  |
| <1990 | 15 | 1.07 (1.03, 1.12) | | 2.3 | 0.43 | 0.07 |  | 13 | 1.15 (1.08, 1.22) | 0 | 0.71 | 0.12 |
| 1990-2000 | 31 | 1.06 (1.00, 1.13) | | 38.6 | 0.02 |  |  | 15 | 1.05 (0.98, 1.11) | 0 | 0.80 |  |
| 2000- | 27 | 1.22 (1.10, 1.35) | | 52.0 | 0.001 |  |  | 4 | 1.10 (0.95, 1.29) | 0 | 0.53 |  |
| **Obese vs normal** |  |  | |  |  |  |  |  |  |  |  |  |
| <1990 | 14 | 1.18 (1.12, 1.24) | | 3.1 | 0.42 | 0.17 |  | 13 | 1.24 (1.10, 1.39) | 41.0 | 0.06 | 0.53 |
| 1990-2000 | 29 | 1.17 (1.11, 1.24) | | 31.4 | 0.06 |  |  | 15 | 1.19 (1.10, 1.28) | 13.4 | 0.30 |  |
| 2000- | 19 | 1.37 (1.17, 1.61) | | 61.3 | 0.0002 |  |  | 4 | 1.42 (1.02, 1.96) | 64.5 | 0.04 |  |
| **Number of cases** |  |  | |  |  |  |  |  |  |  |  |  |
| **Under vs normal** |  |  | |  |  |  |  |  |  |  |  |  |
| <1000 | 5 | 1.16 (0.72, 1.86) | | 0 | 0.75 | 0.75 |  | 2 | 0.90 (0.65, 1.25) | 0 | 0.83 | 0.2 |
| 1000-5000 | 14 | 1.36 (1.17, 1.59) | | 39.5 | 0.06 |  |  | 9 | 1.38 (0.98, 1.93) | 57.7 | 0.02 |  |
| 5000- | 12 | 1.40 (1.21, 1.62) | | 66.4 | 0.0006 |  |  | 5 | 1.11 (0.94, 1.31) | 5.4 | 0.38 |  |
| **Over vs normal** |  |  | |  |  |  |  |  |  |  |  |  |
| <1000 | 20 | 1.06 (0.89, 1.25) | | 44.1 | 0.02 | 0.09 |  | 3 | 0.94 (0.72, 1.22) | 0 | 0.52 | 0.21 |
| 1000-5000 | 37 | 1.15 (1.08, 1.22) | | 47.6 | 0.0008 |  |  | 23 | 1.13 (1.07, 1.19) | 0 | 0.69 |  |
| 5000- | 17 | 1.05 (1.01, 1.10) | | 19.2 | 0.23 |  |  | 6 | 1.06 (0.99, 1.14) | 0 | 0.82 |  |
| **Obese vs normal** |  |  | |  |  |  |  |  |  |  |  |  |
| <1000 | 15 | 1.19 (1.00, 1.41) | | 38.7 | 0.06 | 0.2 |  | 4 | 1.43 (1.01, 2.02) | 65.7 | 0.03 | 0.18 |
| 1000-5000 | 31 | 1.26 (1.17, 1.34) | | 46.0 | 0.003 |  |  | 22 | 1.25 (1.15, 1.35) | 25.4 | 0.14 |  |
| 5000- | 17 | 1.16 (1.10, 1.22) | | 24.8 | 0.17 |  |  | 6 | 1.13 (1.03, 1.24) | 12.6 | 0.33 |  |
| **Mean age** |  |  | |  |  |  |  |  |  |  |  |  |
| **Under vs normal** |  |  | |  |  |  |  |  |  |  |  |  |
| <50 | 4 | 1.24 (0.82, 1.87) | | 66.0 | 0.03 | 0.73 |  | 2 | 0.87 (0.68, 1.12) | 0 | 0.79 | 0.02 |
| 50-55 | 8 | 1.28 (1.02, 1.61) | | 0 | 0.8 |  |  | 0 | — |  |  |  |
| 55- | 9 | 1.44 (1.14, 1.81) | | 72.9 | 0.0003 |  |  | 10 | 1.27 (1.04, 1.56) | 33.8 | 0.14 |  |
| **Over vs normal** |  |  | |  |  |  |  |  |  |  |  |  |
| <50 | 8 | 1.09 (0.94, 1.27) | | 53.8 | 0.034 | 0.34 |  | 2 | 1.00 (0.88, 1.14) | 0 | 0.88 | 0.14 |
| 50-55 | 24 | 1.16 (1.07, 1.25) | | 37.7 | 0.03 |  |  | 6 | 1.19 (1.05, 1.34) | 0 | 0.80 |  |
| 55- | 15 | 1.07 (0.99, 1.15) | | 33.3 | 0.1 |  |  | 14 | 1.07 (1.00, 1.14) | 0 | 0.83 |  |
| **Obese vs normal** |  |  | |  |  |  |  |  |  |  |  |  |
| <50 | 5 | 1.13 (0.97, 1.33) | | 40.3 | 0.15 | 0.56 |  | 2 | 1.12 (0.96, 1.31) | 9.5 | 0.29 | 0.42 |
| 50-55 | 19 | 1.25 (1.16, 1.34) | | 11.4 | 0.32 |  |  | 5 | 1.22 (1.03, 1.46) | 30.1 | 0.22 |  |
| 55- | 12 | 1.24 (1.13, 1.35) | | 34.9 | 0.11 |  |  | 14 | 1.29 (1.13, 1.47) | 49.0 | 0.02 |  |
| **Proportion of obesity** |  |  | |  |  |  |  |  |  |  |  |  |
| **Under vs normal** |  |  | |  |  |  |  |  |  |  |  |  |
| <20 | 10 | 1.24 (1.03, 1.48) | | 29.7 | 0.17 | 0.12 |  | 7 | 1.32 (0.96, 1.81) | 57.6 | 0.03 | 0.57 |
| 20-30 | 6 | 1.68 (1.20, 2.34) | | 73.8 | 0.002 |  |  | 3 | 1.15 (0.83, 1.60) | 0 | 0.64 |  |
| 30- | 0 | — | |  |  |  |  | 1 | 2.61 (1.20, 5.68) |  |  |  |
| **Over vs normal** |  |  | |  |  |  |  |  |  |  |  |  |
| <20 | 19 | 1.09 (1.04, 1.15) | | 10.1 | 0.33 | 0.31 |  | 11 | 1.13 (1.05, 1.22) | 7.9 | 0.37 | 0.37 |
| 20-30 | 13 | 1.03 (0.97, 1.09) | | 0 | 0.61 |  |  | 7 | 1.07 (0.99, 1.15) | 0 | 0.59 |  |
| **Obese vs normal** |  |  | |  |  |  |  |  |  |  |  |  |
| <20 | 19 | 1.34 (1.23, 1.46) | | 41.1 | 0.03 | 0.01 |  | 10 | 1.39 (1.21, 1.58) | 30.5 | 0.17 | 0.07 |
| 20-30 | 15 | 1.19 (1.10, 1.28) | | 30.8 | 0.12 |  |  | 8 | 1.15 (1.05, 1.26) | 14.1 | 0.32 |  |
| 30- | 6 | 1.07 (0.94, 1.22) | | 14.6 | 0.32 |  |  | 3 | 1.13 (0.88, 1.47) | 21.6 | 0.28 |  |
| **Proportion of chemo-therapy** |  |  | |  |  |  |  |  |  |  |  |  |
| **Under vs normal** |  |  | |  |  |  |  |  |  |  |  |  |
| <40 | 2 | 1.30 (0.54, 3.14) | | 79.3 | 0.03 | 0.96 |  | 1 | 0.88 (0.65, 1.19) |  |  |  |
| 40-70 | 5 | 1.36 (1.21, 1.53) | | 0 | 0.42 |  |  | 4 | 1.28 (0.97, 1.68) | 36.5 | 0.19 |  |
| 70- | 6 | 1.31 (0.99, 1.73) | | 0 | 0.87 |  |  | 0 | — |  |  |  |
| **Over vs normal** |  |  | |  |  |  |  |  |  |  |  |  |
| <40 | 7 | 1.14 (1.01, 1.29) | | 43.8 | 0.1 | 0.96 |  | 4 | 1.06 (0.92, 1.22) | 0 | 0.79 | 0.84 |
| 40-70 | 10 | 1.12 (1.03, 1.23) | | 38.0 | 0.11 |  |  | 7 | 1.04 (0.96, 1.13) | 0 | 0.69 |  |
| 70- | 18 | 1.14 (1.04, 1.27) | | 50.8 | 0.007 |  |  | 1 | 1.34 (0.90, 2.00) |  |  |  |
| **Obese vs normal** |  |  | |  |  |  |  |  |  |  |  |  |
| <40 | 4 | 1.20 (1.09, 1.31) | | 0 | 0.63 | 0.43 |  | 4 | 1.40 (1.11, 1.76) | 36.1 | 0.20 | 0.21 |
| 40-70 | 9 | 1.30 (1.18, 1.43) | | 28.6 | 0.19 |  |  | 7 | 1.19 (1.08, 1.32) | 13.9 | 0.32 |  |
| 70- | 12 | 1.21 (1.09, 1.35) | | 42.1 | 0.06 |  |  | 0 | — |  |  |  |
| **Proportion of stage III-IV** |  |  | |  |  |  |  |  |  |  |  |  |
| **Under vs normal** |  |  | |  |  |  |  |  |  |  |  |  |
| <10 | 2 | 1.41 (1.18, 1.69) | | 0 | 0.95 | 0.79 |  | 2 | 1.12 (0.89, 1.43) | 0 | 0.52 |  |
| 10-20 | 9 | 1.35 (1.17, 1.54) | | 0 | 0.52 |  |  | 1 | 1.33 (0.92, 1.92) |  |  |  |
| 20- | 2 | 1.55 (0.998, 2.42) | | 0 | 0.34 |  |  | 0 | — |  |  |  |
| **Over vs normal** |  |  | |  |  |  |  |  |  |  |  |  |
| <10 | 11 | 1.16 (1.02, 1.31) | | 34.4 | 0.12 | 0.35 |  | 5 | 1.05 (0.94, 1.17) | 0 | 0.65 | 0.69 |
| 10-20 | 12 | 1.07 (1.01, 1.13) | | 0 | 0.9 |  |  | 2 | 1.01 (0.89, 1.15) | 6.3 | 0.30 |  |
| 20- | 6 | 1.24 (0.91, 1.70) | | 70.3 | 0.005 |  |  | 1 | 1.34 (0.90, 2.00) |  |  |  |
| **Obese vs normal** |  |  | |  |  |  |  |  |  |  |  |  |
| <10 | 0 | — | |  |  |  |  | 0 | — |  |  |  |
| 10-20 | 10 | 1.22 (1.12, 1.33) | | 9.1 | 0.36 | 0.27 |  | 2 | 1.14 (0.99, 1.32) | 5.1 | 0.30 |  |
| 20- | 4 | 1.06 (0.84, 1.33) | | 39.3 | 0.18 |  |  | 0 | — |  |  |  |

Abbreviations: BSCM, breast cancer specific mortality.

ph1: *P*-value for heterogeneity between studies

ph2: *P*-value from meta-regression

# Supplementary Table S4. Meta-analyses of BMI and outcomes among breast cancer patients

|  | **BMI before diagnosis** | | | |  | | **BMI <1 year post-diagnosis** | | | | |  | | **BMI 1+ year post-diagnosis** | | | | |  |
| --- | --- | --- | --- | --- | --- | --- | --- | --- | --- | --- | --- | --- | --- | --- | --- | --- | --- | --- | --- |
|  | **N** | **RR (95% CI)** | ***I*^2^ (%)** | ***p*_het_** | |  | | **N** | **RR (95% CI)** | ***I*^2^ (%)** | ***p*_het_** | |  | | **N** | **RR (95% CI)** | ***I*^2^ (%)** | ***p*_het_** | |
| **All-cause mortality** |  |  |  |  | |  | |  |  |  |  | |  | |  |  |  |  | |
| Under vs normal | 10 | 1.31 (1.05, 1.64) | 58.3 | 0.01 | |  | | 20 | 1.32 (1.22, 1.43) | 16.2 | 0.25 | |  | | 5 | 1.50 (1.13, 1.99) | 74.7 | 0.003 | |
| Exclusion 1 | 8 | 1.30 (0.99, 1.70) | 64.5 | 0.006 | |  | | 18 | 1.31 (1.20, 1.43) | 21.5 | 0.2 | |  | | 5 | 1.50 (1.13, 1.99) | 74.7 | 0.003 | |
| Exclusion 2 | 10 | 1.31 (1.05, 1.64) | 59.3 | 0.01 | |  | | 17 | 1.31 (1.15, 1.48) | 23 | 0.19 | |  | | 5 | 1.50 (1.13, 1.99) | 74.7 | 0.003 | |
| Over vs normal | 16 | 1.08 (1.01, 1.16) | 20.1 | 0.22 | |  | | 63 | 1.13 (1.08, 1.19) | 44.7 | <0.001 | |  | | 5 | 0.98 (0.90, 1.06) | 0 | 0.63 | |
| Exclusion 1 | 14 | 1.09 (1.10, 1.19) | 30.1 | 0.14 | |  | | 54 | 1.12 (1.07, 1.18) | 44.2 | <0.001 | |  | | 5 | 0.98 (0.90, 1.06) | 0 | 0.63 | |
| Exclusion 2 | 16 | 1.08 (1.01, 1.16) | 20.1 | 0.22 | |  | | 57 | 1.13 (1.07, 1.18) | 41.7 | <0.001 | |  | | 5 | 0.98 (0.90, 1.06) | 0 | 0.63 | |
| Obese vs normal | 18 | 1.28 (1.20, 1.38) | 17.0 | 0.25 | |  | | 53 | 1.21 (1.15, 1.27) | 41.7 | 0.001 | |  | | 6 | 1.10 (0.99, 1.21) | 2.0 | 0.40 | |
| Exclusion 1 | 16 | 1.29 (1.19, 1.39) | 19.1 | 0.24 | |  | | 49 | 1.21 (1.15, 1.27) | 42.4 | 0.001 | |  | | 6 | 1.10 (0.99, 1.21) | 2 | 0.4 | |
| Exclusion 2 | 16 | 1.30 (1.19, 1.42) | 26.2 | 0.16 | |  | | 46 | 1.21 (1.15, 1.28) | 38.5 | 0.005 | |  | | 6 | 1.10 (0.99, 1.21) | 2 | 0.4 | |
| **BCSM** |  |  |  |  | |  | |  |  |  |  | |  | |  |  |  |  | |
| Under vs normal | 6 | 0.94 (0.81, 1.10) | 0 | 0.53 | |  | | 9 | 1.35 (1.11, 1.64) | 35.2 | 0.14 | |  | | 2 | 1.39 (0.81, 2.36) | 0 | 0.81 | |
| Exclusion 1 | 5 | 0.95 (0.81, 1.11) | 2.6 | 0.39 | |  | | 9 | 1.35 (1.11, 1.64) | 35.2 | 0.14 | |  | | 2 | 1.39 (0.81, 2.36) | 0 | 0.81 | |
| Exclusion 2 | 6 | 0.94 (0.81, 1.10) | 0 | 0.53 | |  | | 9 | 1.35 (1.11, 1.64) | 35.2 | 0.14 | |  | | 2 | 1.39 (0.81, 2.36) | 0 | 0.81 | |
| Over vs normal | 11 | 1.06 (0.99, 1.14) | 0 | 0.69 | |  | | 24 | 1.12 (1.07, 1.16) | 0 | 0.53 | |  | | 2 | 1.07 (0.93, 1.24) | 0 | 0.35 | |
| Exclusion 1 | 10 | 1.07 (0.99, 1.15) | 0 | 0.61 | |  | | 23 | 1.12 (1.07, 1.17) | 0 | 0.61 | |  | | 2 | 1.07 (0.93, 1.24) | 0 | 0.35 | |
| Exclusion 2 | 11 | 1.06 (0.99, 1.14) | 0 | 0.69 | |  | | 23 | 1.12 (1.07, 1.17) | 0 | 0.55 | |  | | 2 | 1.07 (0.93, 1.24) | 0 | 0.35 | |
| Obese vs normal | 13 | 1.17 (1.08, 1.27) | 0 | 0.72 | |  | | 27 | 1.22 (1.13, 1.32) | 37.8 | 0.03 | |  | | 2 | 1.49 (0.69, 3.22) | 89.2 | 0.002 | |
| Exclusion 1 | 12 | 1.18 (1.08, 1.29) | 0 | 0.66 | |  | | 26 | 1.22 (1.13, 1.32) | 39.4 | 0.02 | |  | | 2 | 1.49 (0.69, 3.22) | 89.2 | 0.002 | |
| Exclusion 2 | 10 | 1.16 (1.06, 1.26) | 0 | 0.55 | |  | | 26 | 1.24 (1.14, 1.34) | 38 | 0.03 | |  | | 2 | 1.49 (0.69, 3.22) | 89.2 | 0.002 | |
| **Recurrence** |  |  |  |  | |  | |  |  |  |  | |  | |  |  |  |  | |
| Under vs normal | 4 | 1.06 (0.85, 1.31) | 0 | 0.97 | |  | | 11 | 1.39 (1.12, 1.72) | 60.7 | 0.005 | |  | | 1 | 1.17 (0.87, 1.57) |  |  | |
| Exclusion 1 | 3 | 1.05 (0.79, 1.41) | 6.3 | 0.34 | |  | | 9 | 1.42 (1.09, 1.84) | 67.9 | 0.002 | |  | | 1 | 1.17 (0.87, 1.57) |  |  | |
| Exclusion 2 | 4 | 1.06 (0.85, 1.31) | 0 | 0.5 | |  | | 9 | 1.27 (1.04, 1.56) | 46.7 | 0.06 | |  | | 1 | 1.17 (0.87, 1.57) |  |  | |
| Over vs normal | 9 | 1.13 (1.03, 1.24) | 0 | 0. 95 | |  | | 55 | 1.13 (1.08, 1.18) | 44.9 | <0.001 | |  | | 1 | 1.49 (0.98, 2.26) | — | — | |
| Exclusion 1 | 8 | 1.14 (1.03, 1.26) | 0 | 0.93 | |  | | 50 | 1.13 (1.08, 1.19) | 15.6 | 0.19 | |  | | 1 | 1.40 (1.05, 1.86) |  |  | |
| Exclusion 2 | 9 | 1.13 (1.03, 1.24) | 0 | 0.95 | |  | | 48 | 1.12 (1.07, 1.17) | 37.6 | 0.005 | |  | | 1 | 1.49 (0.98, 2.26) |  |  | |
| Obese vs normal | 9 | 1.21 (1.00, 1.45) | 41.4 | 0.09 | |  | | 45 | 1.15 (1.11, 1.19) | 14.4 | 0.21 | |  | | 1 | 1.40 (1.05, 1.86) | — | — | |
| Exclusion 1 | 8 | 1.18 (0.96, 1.45) | 44.3 | 0.08 | |  | | 42 | 1.15 (1.10, 1.19) | 15.6 | 0.19 | |  | | 1 | 1.40 (1.05, 1.86) |  |  | |
| Exclusion 2 | 9 | 1.21 (1.00, 1.45) | 41.4 | 0.09 | |  | | 40 | 1.15 (1.10, 1.20) | 18.8 | 0.15 | |  | | 1 | 1.40 (1.05, 1.86) |  |  | |

Abbreviations: BMI, body mass index; BCSM, breast cancer specific mortality; RR, relative risk.

Exclusion 1: studies with metastatic cases

Exclusion 2: high risk of bias (NOS <5) or unclear risk of bias

P_het_: P-value for heterogeneity between studies

# Supplementary Figure S1. Meta-analysis of BMI before diagnosis and all-cause mortality

#
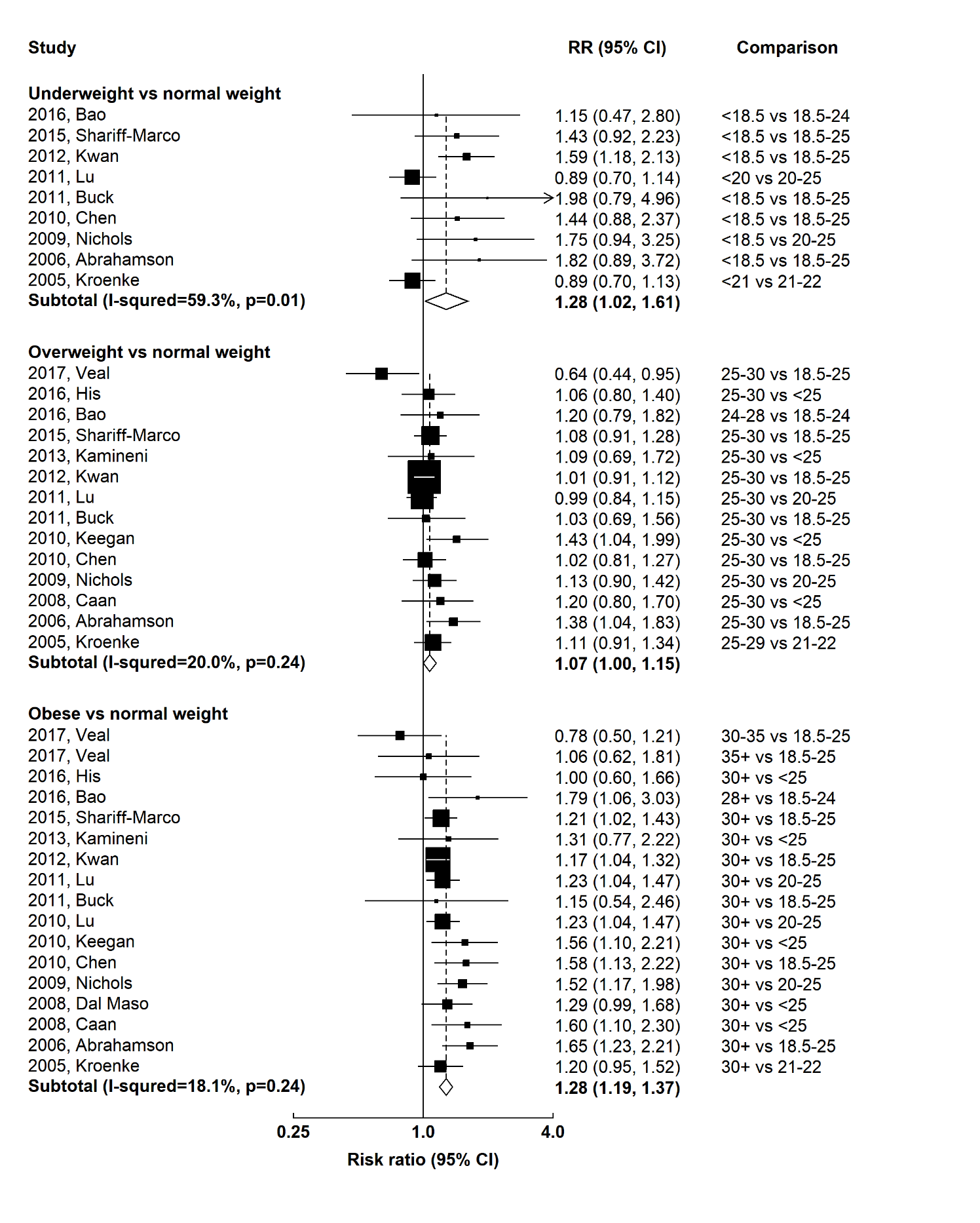


# Supplementary Figure S2. Meta-analysis of BMI before diagnosis and BCSM


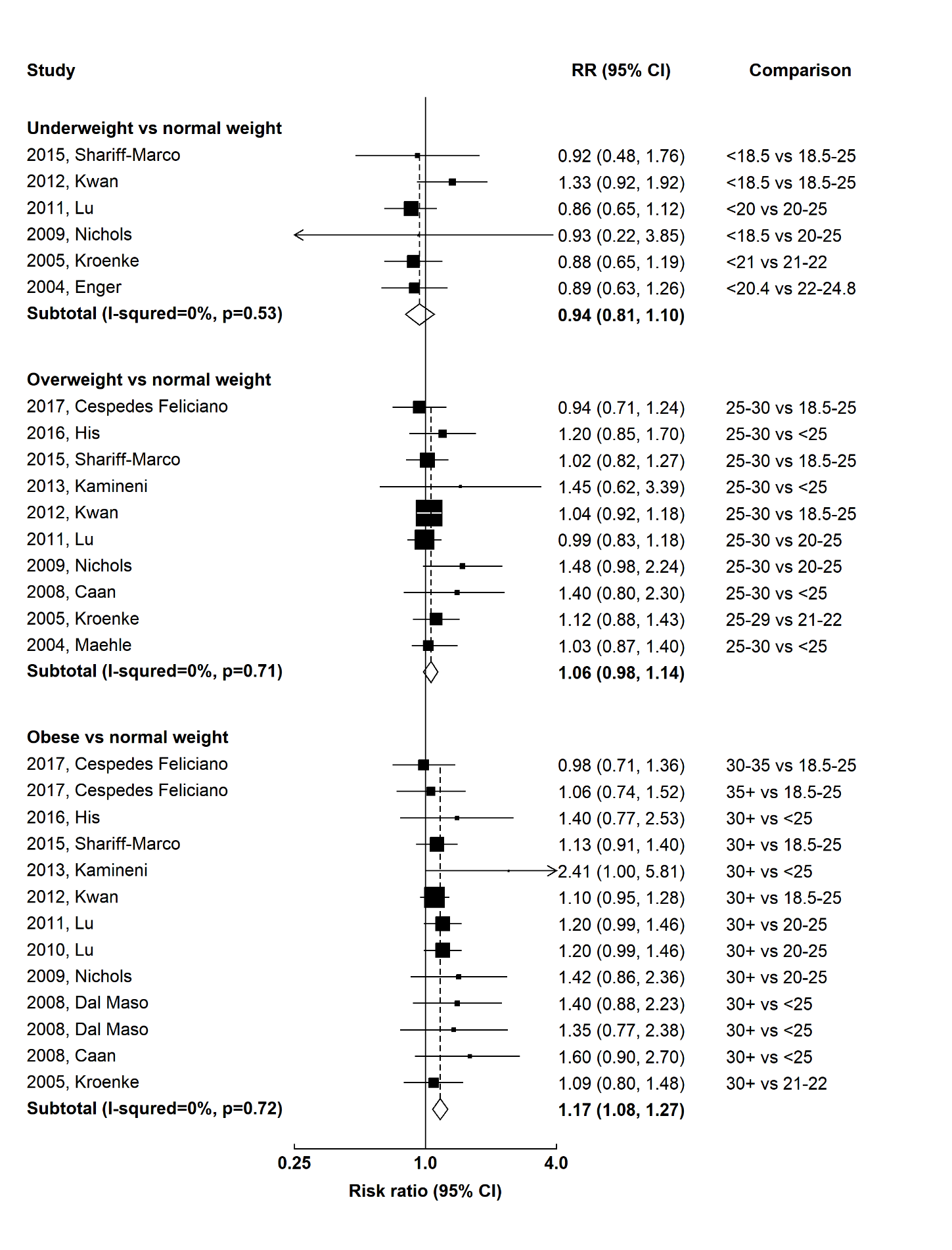


# Supplementary Figure S3. Meta-analysis of BMI before diagnosis and recurrence


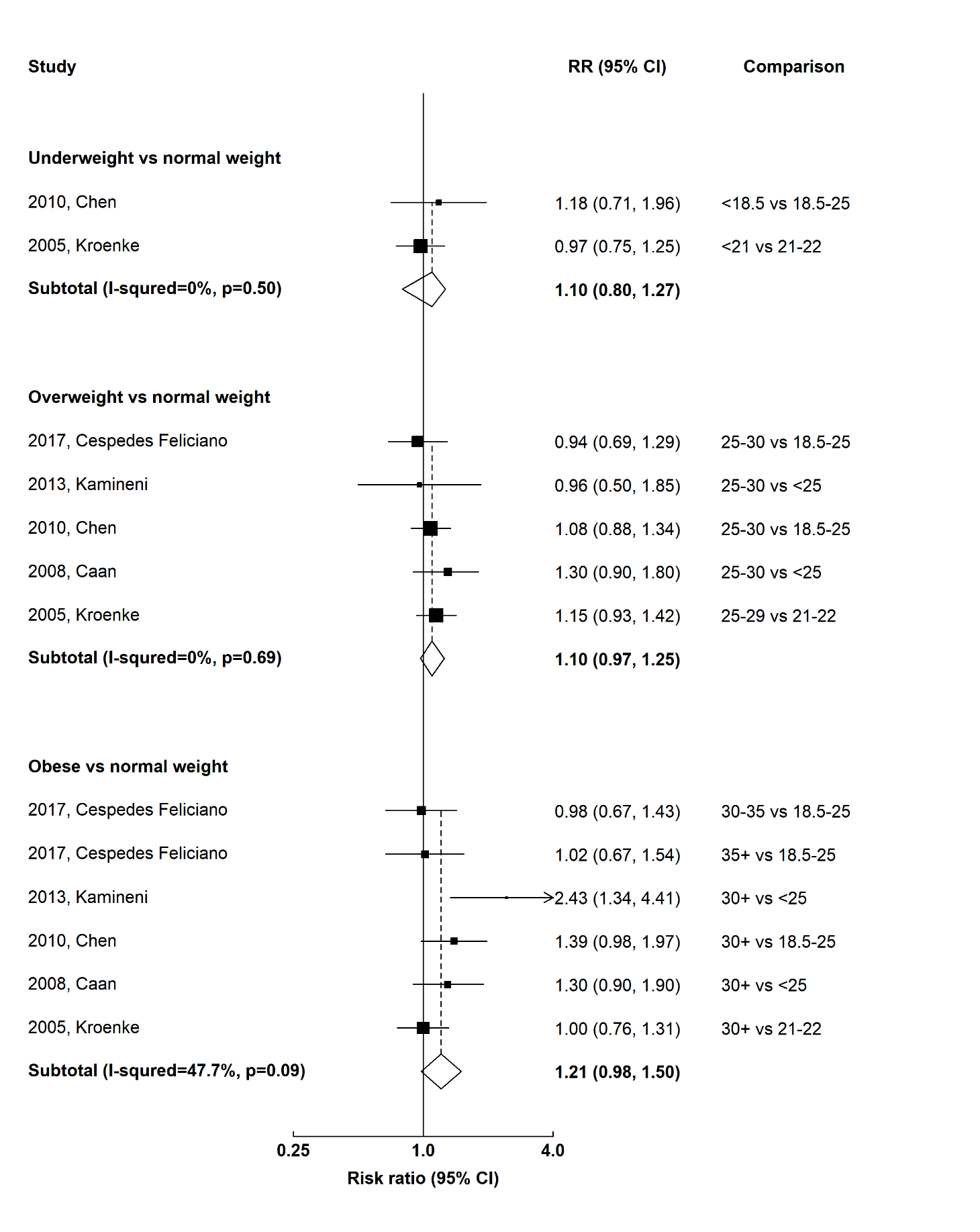


# Supplementary Figure S4. Funnel plot

**
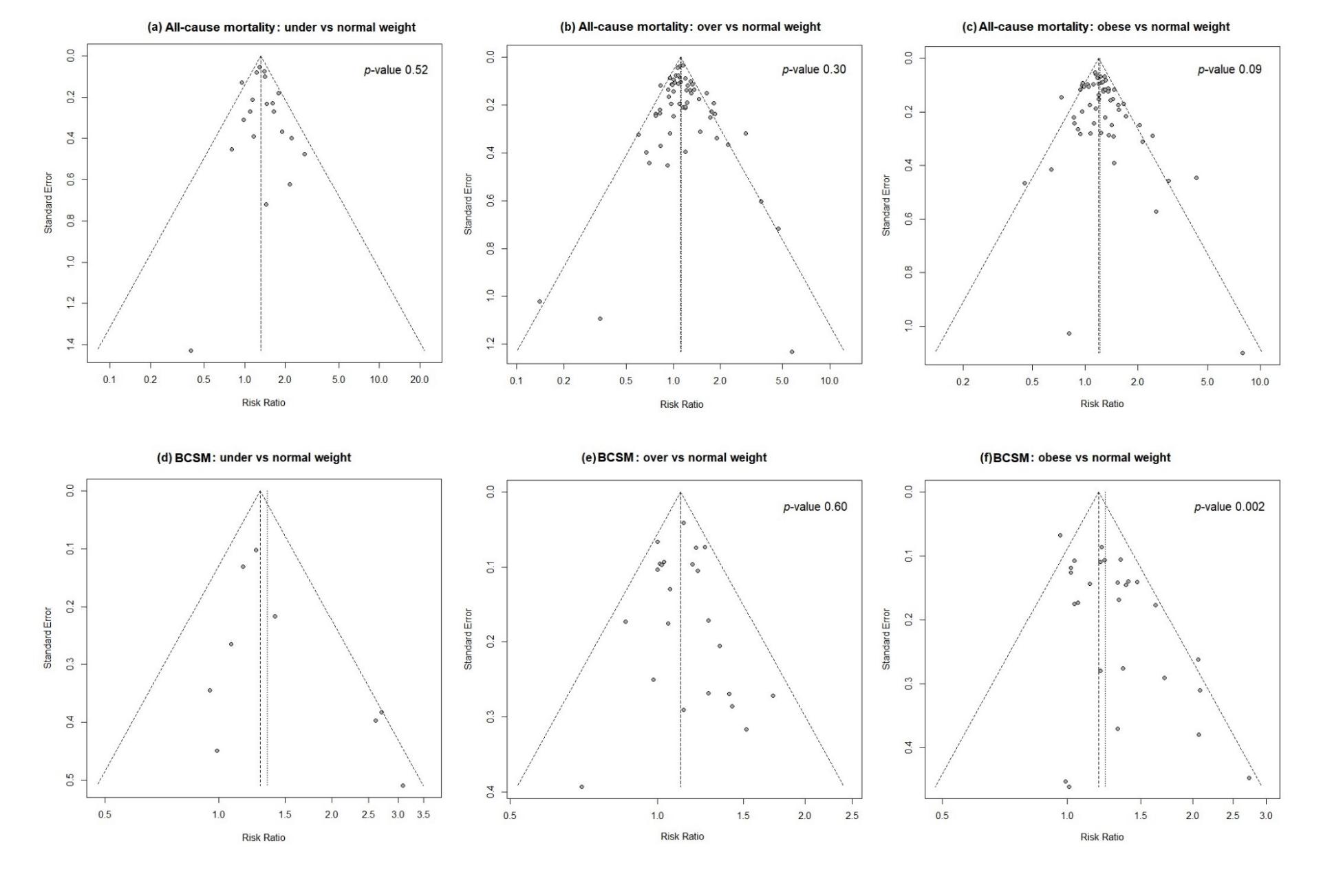
**

# PROSPERO Protocol

**Citation**

Christiana Kartsonaki, Yuanjie Pang, Yuxia Wei. Associations of adiposity and weight change with recurrence and survival in breast cancer patients. PROSPERO 2020 CRD42020214730 Available from: <https://www.crd.york.ac.uk/prospero/display_record.php?ID=CRD42020214730>

**Searches**

Databases to be searched:

- PubMed (Ovid)

- EMBASE (Ovid)

Searches will include articles up to August 2020.

In addition we will search reference lists of included studies.

We will not apply language restrictions to any of the searches.

Searches will be re-run prior to the final analysis and any other studies identified will be retrieved for inclusion.

**Types of study to be included**

Inclusions:

1. Prospective cohort studies

2. RCTs or other non-randomised trials

3. Case series with more than 50 patients

Exclusions:

1. Retrospective studies

2. Reviews

3. Conference/poster abstracts, where sufficient data cannot be obtained

4. Studies on patients with inoperable or metastatic breast cancer

If multiple articles were published from the same study, we will use the one with the largest number of patients.

**Condition or domain being studied**

Early or locally advanced operable breast cancer.

**Participants/population**

Adults (18+ years of age) with early or operable locally advanced breast cancer who receive treatment with curative intent.

**Intervention(s), exposure(s)**

General adiposity (body mass index), weight change, or weight loss interventions, measured or performed immediately prior to, during or after treatment.

**Comparator(s)/control**

Not applicable.

**Main outcome(s)**

Breast cancer-specific survival, overall survival, distant metastasis-free survival.

**** Measures of effect***

Hazard ratios/relative risks

**Additional outcome(s)**

Time to isolated local/regional recurrence, disease-free survival.

**** Measures of effect***

Hazard ratios/relative risks

**Data extraction (selection and coding)**

The titles and/or abstracts identified by the search will be screened by one author. Duplicates and articles which do not meet the inclusion criteria will be removed.

The full texts of all studies identified as being potentially eligible for inclusion will then be obtained and assessed by two review authors, who will independently screen and assess their eligibility for inclusion. Disagreements will be addressed by discussion between the two authors, with any remaining differences being resolved by recourse to a third review author. Data will be extracted and stored in a data extraction form. A second author will review the data extraction.

The following information will be extracted from studies meeting the selection criteria:

- Citation details

- Study design (e.g. RCT, observational cohort study) and methodology details

- Study population

- Number of participants

- Years during which participants were recruited

- Duration of follow-up

- Exposure (for observational studies) or intervention type and details (including adherence to intervention) (for RCTs)

- Reference group (for observational studies) or control (for RCTs)

- Measured outcomes

- Methods of measurement of exposure(s) and outcome(s)

- Confounders considered (for observational studies)

- Estimates of association/effect

- Numbers and estimates of association by subgroup:

- By menopause status at diagnosis

- By diagnosis methods (screen-detected or symptomatic)

- By molecular subtype (ER+ vs ER-, or ER+/HER2-, HER2+, triple negative, or luminal A, luminal B, HER2 enriched, basal)

- By grade

- By stage (or local/regional, or lymph node involvement/no involvement)

- By treatment (or in subgroups of treatment), e.g. among participants treated with aromatase inhibitors

- Country where study was performed

- Characteristics of participants: age (mean/median/range), sex, race/ethnicity, proportions by grade, stage and molecular subtype

**Risk of bias (quality) assessment**

For randomised studies, we will evaluate the risk of bias based on the Cochrane Collaboration ‘Risk of bias’ tool (‘high risk of bias’, ‘low risk of bias’, or ‘unclear’). For nonrandomised studies, we will use a modified Newcastle-Ottawa quality-assessment scale, including eight items with nine scores. This scale will be used to assess the adequacy in selection of participants (four scores), comparability (two scores), and outcomes (three scores). All included studies will be accessed by two authors (YP and YW) independently. Discrepancies will be resolved through discussion.

We will summarise judgements in risk of bias tables. Results of meta-analyses will be interpreted with reference to the overall risk of bias assessment. Publication bias will be assessed using funnel plots, Egger’s test and ‘trim and fill’.

**Strategy for data synthesis**

A table and descriptive summary will be generated for all included studies. We will perform both a fixed-effect inverse-variance weighted and a random-effects meta-analysis.

- Overweight and obese vs normal weight

- Dose-response meta-analysis (using the method described by Greenland and Longnecker)

- Weight change

Results will be presented graphically using forest plots. Heterogeneity will be assessed using the I² statistic. Analysis will be done using R and package ‘meta’ and ‘dosresmeta’.

**Analysis of subgroups or subsets**

Subject to availability of reported estimates we will investigate associations in subgroups of patients or subgroups of studies as follows:

1. Subgroups by menopause status or meta-regression by proportion pre- or post-menopausal for overall estimates

2. Subgroups by subtypes or meta-regression by proportions of subtypes, grade, stage

3. Meta-regression by time when study was performed/patients were recruited (to investigate any association is modified by change in treatments, screening, detection of recurrence)

4. Subgroups or meta-regression by treatment (receipt of chemotherapy and among ER+ subtypes receipt of SERM/aromatase inhibitors)

5. Subgroups by ethnicity or meta-regression by ethnicity composition

6. Subgroups by method of detection (screening/symptoms) or meta-regression by proportions screen-detected/symptomatic

7. Meta-regression by mean age at diagnosis

8. If any studies identified are in special populations, such as in germline BRCA1/2 carriers, or include patients with ductal carcinoma in situ
